# Identification and localization of *Tospovirus* genus-wide conserved residues in 3D models of the nucleocapsid and the silencing suppressor proteins

**DOI:** 10.1186/s12985-018-1106-4

**Published:** 2019-01-11

**Authors:** Cristian Olaya, Badri Adhikari, Gaurav Raikhy, Jianlin Cheng, Hanu R. Pappu

**Affiliations:** 1 Department of Plant Pathology, Washington State University, Pullman, WA 99164 USA; 2 Department of Mathematics and Computer Science, University of Missouri, St. Louis, MO 63121 USA; 3 Department of Microbiology and Immunology, Louisiana State University, Shreverport, LA 71101 USA; 4 Department of Electrical Engineering and Computer Science, University of Missouri, Columbia, MO 65211 USA

**Keywords:** Conserved amino acids, Tospovirus, Nucleoprotein, Silencing suppressor, Three-dimensional protein structure

## Abstract

**Background:**

Tospoviruses (genus *Tospovirus*, family *Peribunyaviridae*, order *Bunyavirales*) cause significant losses to a wide range of agronomic and horticultural crops worldwide. Identification and characterization of specific sequences and motifs that are critical for virus infection and pathogenicity could provide useful insights and targets for engineering virus resistance that is potentially both broad spectrum and durable. *Tomato spotted wilt virus* (TSWV), the most prolific member of the group, was used to better understand the structure-function relationships of the nucleocapsid gene (N), and the silencing suppressor gene (NSs), coded by the TSWV small RNA.

**Methods:**

Using a global collection of orthotospoviral sequences, several amino acids that were conserved across the genus and the potential location of these conserved amino acid motifs in these proteins was determined. We used state of the art 3D modeling algorithms, MULTICOM-CLUSTER, MULTICOM-CONSTRUCT, MULTICOM-NOVEL, I-TASSER, ROSETTA and CONFOLD to predict the secondary and tertiary structures of the N and the NSs proteins.

**Results:**

We identified nine amino acid residues in the N protein among 31 known tospoviral species, and ten amino acid residues in NSs protein among 27 tospoviral species that were conserved across the genus. For the N protein, all three algorithms gave nearly identical tertiary models. While the conserved residues were distributed throughout the protein on a linear scale, at the tertiary level, three residues were consistently located in the coil in all the models. For NSs protein models, there was no agreement among the three algorithms. However, with respect to the localization of the conserved motifs, G^18^ was consistently located in coil, while H^115^ was localized in the coil in three models.

**Conclusions:**

This is the first report of predicting the 3D structure of any tospoviral NSs protein and revealed a consistent location for two of the ten conserved residues. The modelers used gave accurate prediction for N protein allowing the localization of the conserved residues. Results form the basis for further work on the structure-function relationships of tospoviral proteins and could be useful in developing novel virus control strategies targeting the conserved residues.

**Electronic supplementary material:**

The online version of this article (10.1186/s12985-018-1106-4) contains supplementary material, which is available to authorized users.

## Background

Tospoviruses constitute one of the plant-infecting families in the order *Bunyavirales*, one of the largest and most diverse RNA virus orders, with more than 350 named isolates [1–4]. The order *Bunyavirales* currently consists of ten families: *Arenaviridae*, *Cruliviridae*, *Fimoviridae*, *Hantaviridae*, *Mypoviridae*, *Nairoviridae*, *Peribunyaviridae*, *Phasmaviridae*, *Phenuiviridae* and *Wupedeviridae* (please refer to the International Committee on Taxonomy of Viruses -ICTV- website talk.ictvonline.org for current virus taxonomy) [1]. Tospoviruses are transmitted by thrips [3]; with a broad host range of more than 1000 plant species, these viruses infect economically important crops such as bean, pepper, potato, soybean, tobacco and tomato worldwide [5], causing an estimated annual loss of over USD 1 billion globally [4, 6]. Members of the genus *Tospovirus* are characterized by three-segmented, mostly negative sense RNA genomes, named according to size: L (large), M (medium), and S (small) [7]. The L segment encodes an RNA-dependent RNA polymerase (RdRp) in the viral complementary sense orientation; the M, the precursors to glycoproteins G_N_ and G_C_ in the virion complementary sense and the movement protein NSm in the virion sense orientation; and the S, the silencing suppressor protein NSs in the virion sense and the nucleocapsid protein N in the virion complementary sense [6]. The N protein functions as a protective layer encapsidating the three viral genomic RNA segments. But also, plays a role in viral RNA transcription and replication [8].

Recently, non-structural proteins encoded by tospoviruses have received much attention due to their ability to interact with the vector/host immune system and to contribute to the viral pathogenesis. The NSm serves as the movement protein and the NSs has been shown to be the silencing suppressor [9–11]. In plants, accumulation of the TSWV NSs protein has been observed in infected leaves [12]. Furthermore, accumulation of high levels of NSs in the salivary glands of thrips could be indicative of NSs protein being co-injected into plants during thrips feeding [13]. The silencing suppressor proteins of TSWV and Tomato yellow ring virus (TYRV) interfere with the RNA silencing response in plants [14, 15]. However, not all tospoviral NSs proteins have the same affinity for different types of dsRNA molecules [15]. The NSs proteins of the American clade tospoviruses [e.g. TSWV, *Groundnut ring spot virus* (GRSV) and *Impatiens necrotic spot virus* (INSV)] can bind to long and short dsRNA molecules with a similar affinity, while the Eurasian clade NSs (TYRV) can only bind to short dsRNA molecules [15]. A similar variation among viruses of the same genus has been reported for *Tombusvirus* genus. Recently, the NSs of TSWV has been reported as an avirulence (Avr) determinant in pepper (*Capsicum annuum*) [16]. This suggests an additional role for the NSs of TSWV besides the well-defined RNAi suppressor activity. Likewise, it has recently been suggested that the NSs of TSWV has a role in translation [17], and persistent infection and transmission by *Frankliniella occidentalis* [18]. It has been shown that some conserved motifs in tospovirus NSs proteins are essential for its silencing suppressor activity [19–21] and for the helicase and NTPase/phosphatase activity of the NSs of *Groundnut bud necrosis virus* (GBNV; [22, 23]). More research is needed to investigate if the different affinities for the small RNAs observed for the American and Eurasian clades can be associated, for example, with virulence and/or translational activity.

Several regions of the N and NSm have been found to interact with each other [24–28]. Bag et al. [29] found in plants doubly infected with *Irish yellow spot virus* (IYSV) and TSWV, increased titers of N and NSs proteins of IYSV in younger, uninoculated leaves of IYSV-infected plants. It was not clear if the NSs protein modulated the host machinery by suppressing its defense or if there was an enhanced virus assembly and replication due to the interaction of tospovirus proteins (IYSV and TSWV). While much is known about the genome structure, organization and functions of orthotospoviral proteins, little is known of their structure. The protein structure prediction could aid in developing functional hypotheses about hypothetical proteins, improving phasing signals in crystallography, selecting sites for mutagenesis, and designing novel, targeted therapies. Template-based homology modeling or fold-recognition is the most successful approach for predicting the structure of proteins. This approach is based on using homologs of already known three-dimensional (3D) protein structures. This method relies on the observation that the number of folds in nature appears to be limited and that many different remotely homologous protein sequences adopt remarkably similar structures. Thus, one may compare a protein sequence of interest with the sequences of proteins with experimentally determined structures [30]. If a homolog (template) can be found, an alignment of the two sequences can be generated and used directly to build a 3D model of the sequence of interest.

In *Bunyavirales*, structures of virally coded proteins of certain viruses in the genus *Orthobunyavirus* were determined [31–33]. Among tospoviral proteins, the glycoproteins [34] and the N protein of TSWV and GRSV have been predicted by folding prediction [8, 35], but only the N protein structure of TSWV has been determined by crystallization [36–38]. Li et al. [8] have simulated the 3D structure and mapped the RNA binding sites. While the crystal structure of silencing suppressor proteins of a few plant viruses, such as p19 of *Carnation Italian ringspot virus* (CIRV) [39]; p19 of *Tomato bushy stunt virus* (TBSV) [40]; and p2b of *Tomato aspermy virus* (TAV) [41] are available, however, no such information is available for the NSs of any tospovirus.

The objectives of this study were to first identify conserved motifs in N and NSs proteins across the *Tospovirus* genus and determine their potential location on the 3D models of these two proteins of TSWV based on their primary amino acid sequences. Knowledge about the localization of critical amino acid residues could form the basis for further work on the structure-function relationships of tospoviral proteins and could be useful in developing novel, targeted virus control strategies.

## Methods

### Multiple sequence alignments of N and NSs proteins

A total of 31 complete N gene sequences from tospoviruses available in GenBank (Table 1) were used to conduct multiple alignments (MSA) using Clustal W algorithms in MEGA 6.06 software [42] and identify the conserved residues. The complete NSs gene sequences of 27 *Tospovirus* species available in GenBank were used to conduct MSA using Clustal W. Based on MSA, family-wide conserved residues were identified. The output of the MSA were prepared using ESPript 3.0 server [43].

**Table 1:** List of Tospovirus species used to align the nucleocapsid (N) and the non-structural protein coded by the small RNA (NSs) proteins

	Name	Acronym	N protein	NSs protein
1	Alstroemeria necrotic streak virus	ANSV	GQ478668.1	–
2	Bean necrotic mosaic virus	BeNMV	JN587268.1	NC 018071.1
3	Calla lily chlorotic spot virus	CCSV	AY867502.1	AY867502.1
4	Capsicum chlorosis virus	CaCV	NC 008301.1	DQ355974.1
5	Chrysanthemum stem necrosis virus	CSNV	AF067068.1	KM114548.1
6	*Groundnut bud necrosis virus* (Synonymous: Peanut bud necrosis virus)	GBNV	U27809.1	AB852525.1
7	Groundnut chlorotic fan- spot virus (Synonymous: Peanut chlorotic fan-spot virus)	GCFSV	AF080526.1	U27809.1
8	*Groundnut ringspot virus*	GRSV	AF251271.1	AF080526.1
9	*Groundnut yellow spot virus* (Synonymous: Peanut yellow spot virus)	GYSV	AF013994.1	JN571117.1
10	Hippeastrum chlorotic ringspot virus	HCRV	KC290943.1	AF013994.1
11	*Impatiens necrotic spot virus*	INSV	X66972.1	KC290943.1
12	*Iris yellow spot virus*	IYSV	AF001387.1	AB109100.1
13	Lisianthus necrotic ringspot virus	LNRV	AB852525.1	AF001387.1
14	Melon severe mosaic virus	MSMV	EU275149.1	EU275149.1
15	Melon yellow spot virus	MYSV	AB038343.1	AB038343.1
16	Mulberry vein banding associated virus	MVBaV	NC 026619.1	NC 026619.1
17	Pepper chlorotic spot virus	PCSV	KF383956.1	KF383956.1
18	Pepper necrotic spot virus	PNSV	HE584762.1	HE584762.1
19	Physalis silver mottle virus	PhySMV	AF067151.1	AF067151.1
20	*Polygonum ringspot virus*	PolRSV	KJ541744.1	EF445397.1
21	Soybean vein necrosis associated virus	SVNaV	HQ728387.1	HQ728387.1
22	Tomato chlorotic spot virus	TCSV	AF282982.1	–
23	Tomato necrosis virus	TNeV	AY647437.1	–
24	Tomato necrotic ringspot virus	TNRV	FJ489600.2	FJ489600.2
25	Tomato necrotic spot virus	TNSV	KM355773.1	–
26	*Tomato spotted wilt virus*	TSWV	AF020659.1	–
27	Tomato yellow ring virus	TYRV	AY686718.1	DQ462163.1
28	Tomato zonate spot virus	TZSV	EF552433.1	EF552433.1
29	*Watermelon bud necrosis virus*	WBNV	GU584184.1	EU249351.1
30	*Watermelon silver mottle virus*	WSMoV	AB042650.1	AY864852.1
31	*Zucchini lethal chlorosis virus*	ZLCV	AF067069.1	JN572104.1

### Structure prediction of the N protein

Three-dimensional models of the N and the NSs proteins of TSWV were predicted in silico using state-of-the-art protein structure prediction methods, ROSETTA [44], I-TASSER (Iterative Threading ASSEmbly Refinement) [45–47], and the three MULTICOM servers including MULTICOM-CONSTRUCT [48], MULTICOM-CLUSTER [49], and MULTICOM-NOVEL [50]. We used ROSETTA, I-TASSER and MULTICOM web servers [51–53] to predict five models from each of the methods. These methods are ranked as top predictors in the Eleventh Critical Assessment of Protein Structure Prediction (CASP) competitions [54, 55]. The 15 models predicted by MULTICOM servers (3 from each method) were compared pairwise and ranked using APOLLO [56] to obtain the top five models. APOLLO ranks the models based on the average pairwise template modeling score (TM-score) [57], max-sub score, Global-distance test (GDT-TS) score and Q-score [58]. Finally, the top five models from the three sets, each from the MULTICOM servers, ROSETTA, and I-TASSER were compared and ranked by the model quality assessment technique, Qprob. As a single-model quality assessment tool, where, top ranking models score is more than 0.5 represents the best possible common model predicted by all three models [59] (Table 2).

**Table 2:** Qprob score rank of *Tomato spotted wilt virus* (TSWV) nucleocapsid protein, N

TSWV N Comparison between models	Score
TSWV-N-I-TASSER Model 3	0.55
TSWV-N-ROSETTA Model 5	0.549
TSWV-N-ROSETTA Model 1	0.547
TSWV-N-I-TASSER Model 2	0.541
TSWV-N-ROSETTA Model 3	0.54
TSWV-N-I-TASSER Model 5	0.537
TSWV-N-I-TASSER Model 4	0.532
TSWV-N-I-TASSER Model 1	0.531
TSWV-N-ROSETTA Model 2	0.528
TSWV-N-ROSETTA Model 4	0.499
TSWV-N-MULTICOM-Model 3	0.496
TSWV-N-MULTICOM-Model 4	0.496
TSWV-N-MULTICOM-Model 1	0.476
TSWV-N-MULTICOM-Model 2	0.476
TSWV-N-MULTICOM-Model 5	0.44

**Fig. 1:**
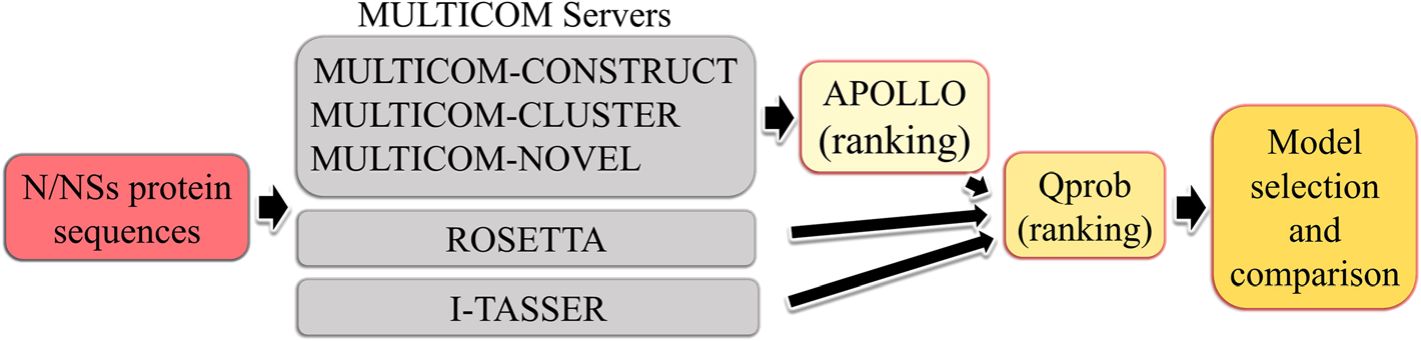
Flow chart showing the steps involved in predicting the 3D models for TSWV (N) and non-structural (NSs) protein sequences

As shown in Fig. 1, this approach was applied to both N and NSs protein sequence to generate models for analysis. Models were visualized using UCSF Chimera version 1.10.1 [60].

### Structure prediction of the NSs protein

We used the same protein structure prediction tools, ROSETTA, I-TASSER and MULTICOM, to predict 3D structures for the NSs protein sequence. For this protein, we found no agreement between the 3D models generated by the three servers. Hence, we resorted to residue-residue contact guided modeling options to predict the structure for the NSs protein sequence. The contact-guided structure prediction methods in the CASP11 [61–63] competition has enabled us to build 3D models by making use of predicted residue-contacts.

The principle of contact-guided protein folding is to predict residue-residue contacts (2D information) first and then utilize this information along with secondary structure prediction (helix, coil and beta-sheet information) to predict tertiary structure (3D) models. The most successful contact prediction methods use machine learning and coevolution information from multiple sequence alignments to predict contacts [64]. Highly confident predicted contacts strongly suggest which residues should be close to each other in the 3D model and many of these predicted pairs together suggest an overall fold of the protein. Many protein modeling tools like ROSETTA, FRAGFOLD, CONFOLD and EVFOLD take these predicted contacts and predicted secondary structure and optimize 3D models for best contact satisfaction score. The confidence of each predicted pair of contacts plays a crucial role for the optimization process. In this paper we chose CONFOLD for modeling because of its speed and free availability.

The NSs protein sequence is relatively long (467 residues) and its structure turned out to be hard to predict because (*i*) there are no templates for this sequence in the PDB database, and (*ii*) there are no more than a few hundred homologous sequences in the sequence databases. When the sequence of protein, whose structure is being predicted, is long (for example, more than 250 residues) and the structure is hard to predict, very often, domain boundaries are predicted to split the sequence into domains and predictions are made for individual domains instead of the whole sequence [65]. Ideally, the next step is to combine the predicted domain models to make a single 3D model, but combining predicted domains is a much harder problem, and hence it is a common practice to study and evaluate the domains separately as in the CASP competitions [55]. For this reason, we used predictions from a state-of-the-art domain boundary prediction tool, DoBo [66], to split the NSs protein sequence into two domains. DoBo predicted a domain boundary at position 254 with 81% confidence. To verify this accuracy, we also submitted the domain boundary prediction job to the ThreaDom web server [65].

**Fig. 2:**
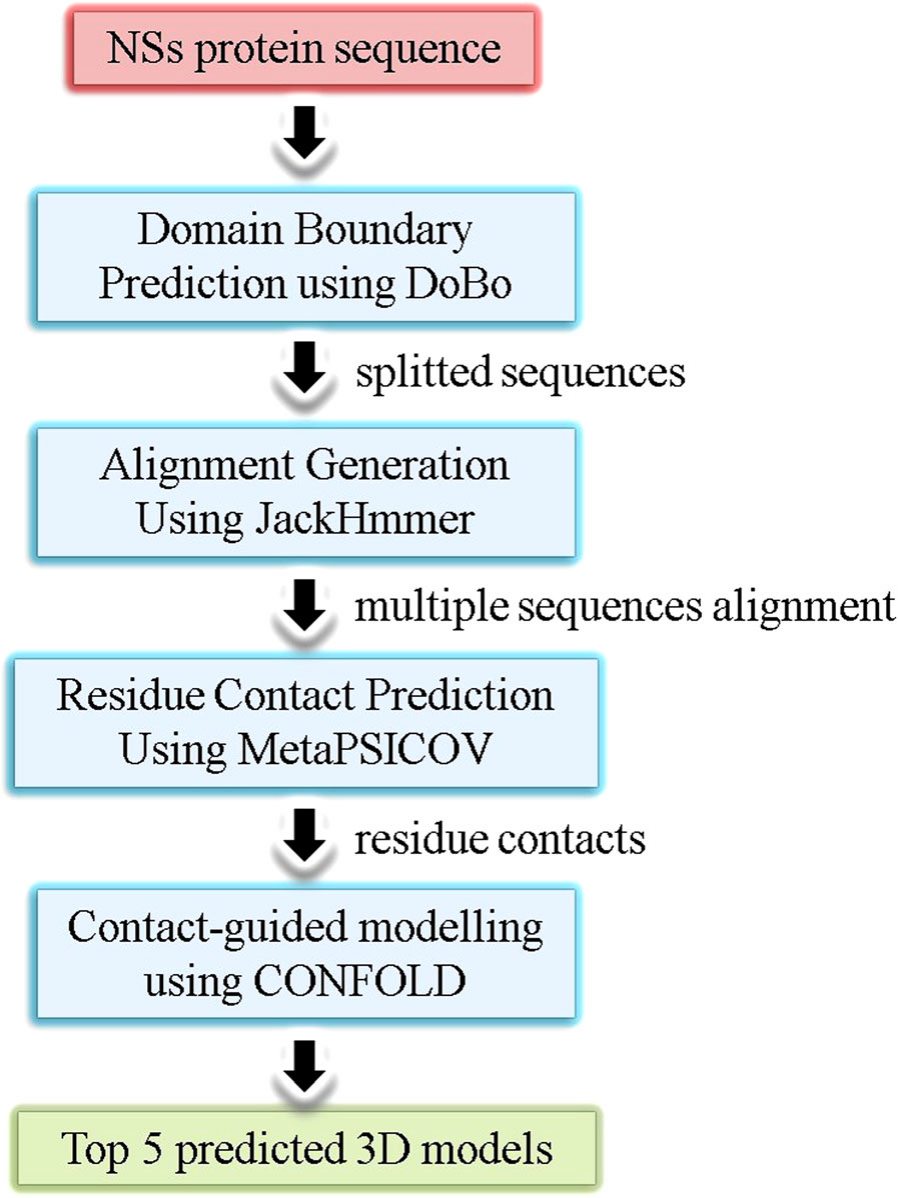
Flow chart showing the steps involved in predicting 3D models for the TSWV non-structural (NSs) protein sequence, using contact-guided ab initio structure prediction tool

After the domain splitting, we had two sequences to predict structures for – domain-I of 254 residues, and domain-II of 213 residues. Then we used, MetaPSICOV [64], the state-of-the-art residue contact prediction tool, to make contact predictions for the two sequences using JackHammer [67] for constructing the MSA. These predicted contacts along with the predicted PSIPRED [68] secondary structures and beta-sheet pairing predicted using BETApro [41], were provided as input to a recently published contact-guided ab initio structure prediction tool CONFOLD [69]. For each of the two sequences, CONFOLD produced five models as final set of models using top 0.8 L, 1 L, 2 L, 3 L, and 4 L predicted contacts, where L is the length of the sequence. We use these ten models (five for each domain) as final predicted 3D models. The approach described above is summarized in Fig. 2 and a list of all programs used is compiled in Additional file 1 : Table S1.

## Results

**Fig. 3:**
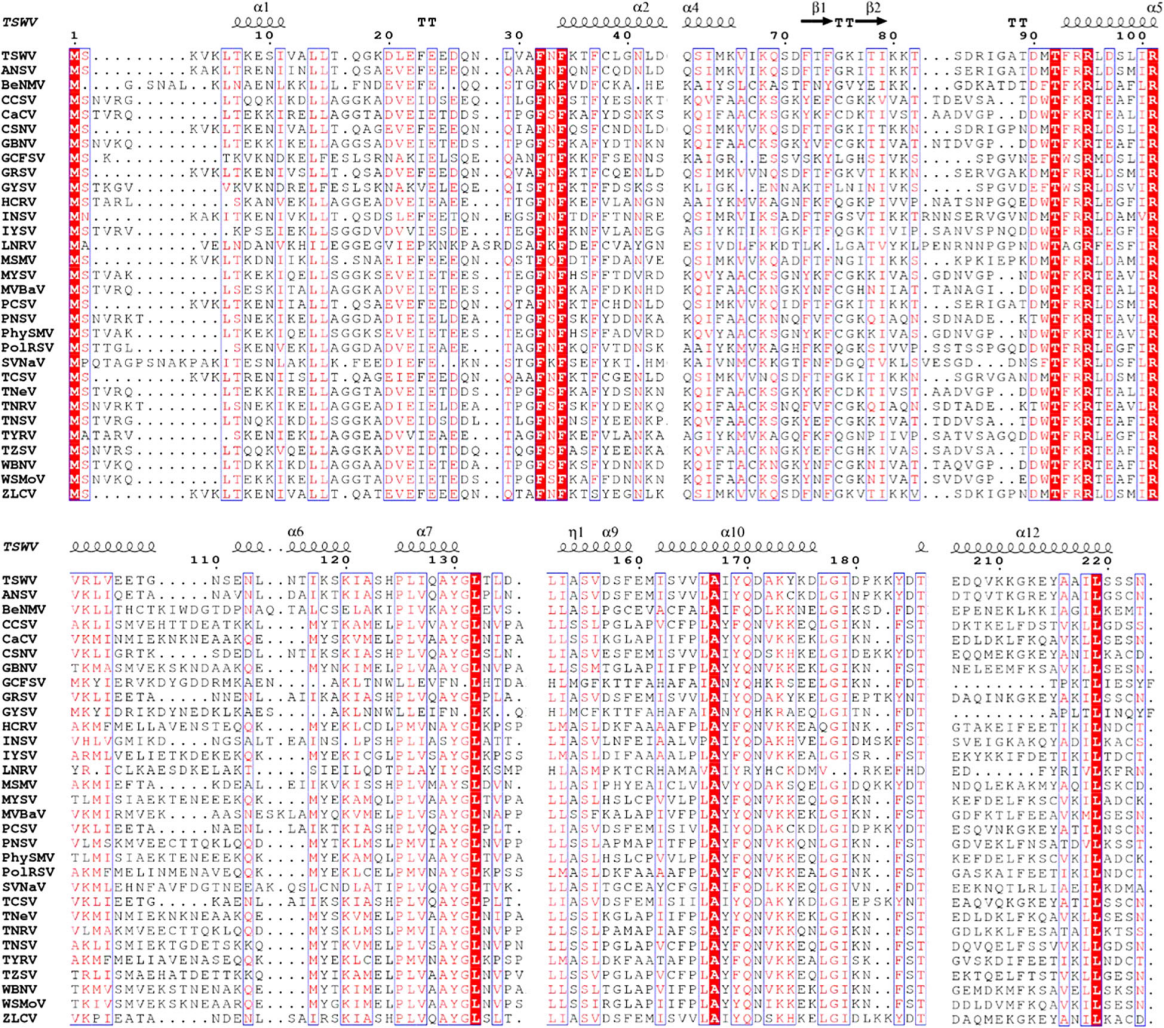
Alignment of the amino acid sequences of the nucleocapsid protein of all known tospoviruses. The list of tospoviruses used is given in Table 1. The columns highlighted in red indicate amino acid residues conserved among all known tospoviruses. The secondary structure of TSWV predicted by I-TASSER is shown above the alignment with arrows and squiggles indicating beta sheets and alpha helices, respectively. Amino acid residues conserved among all known tospoviruses are indicated in red. The figure was prepared using ESPript 3.0 server [40]. An extended version can be find in Additional file 2: Figure S1

**Fig. 4:**
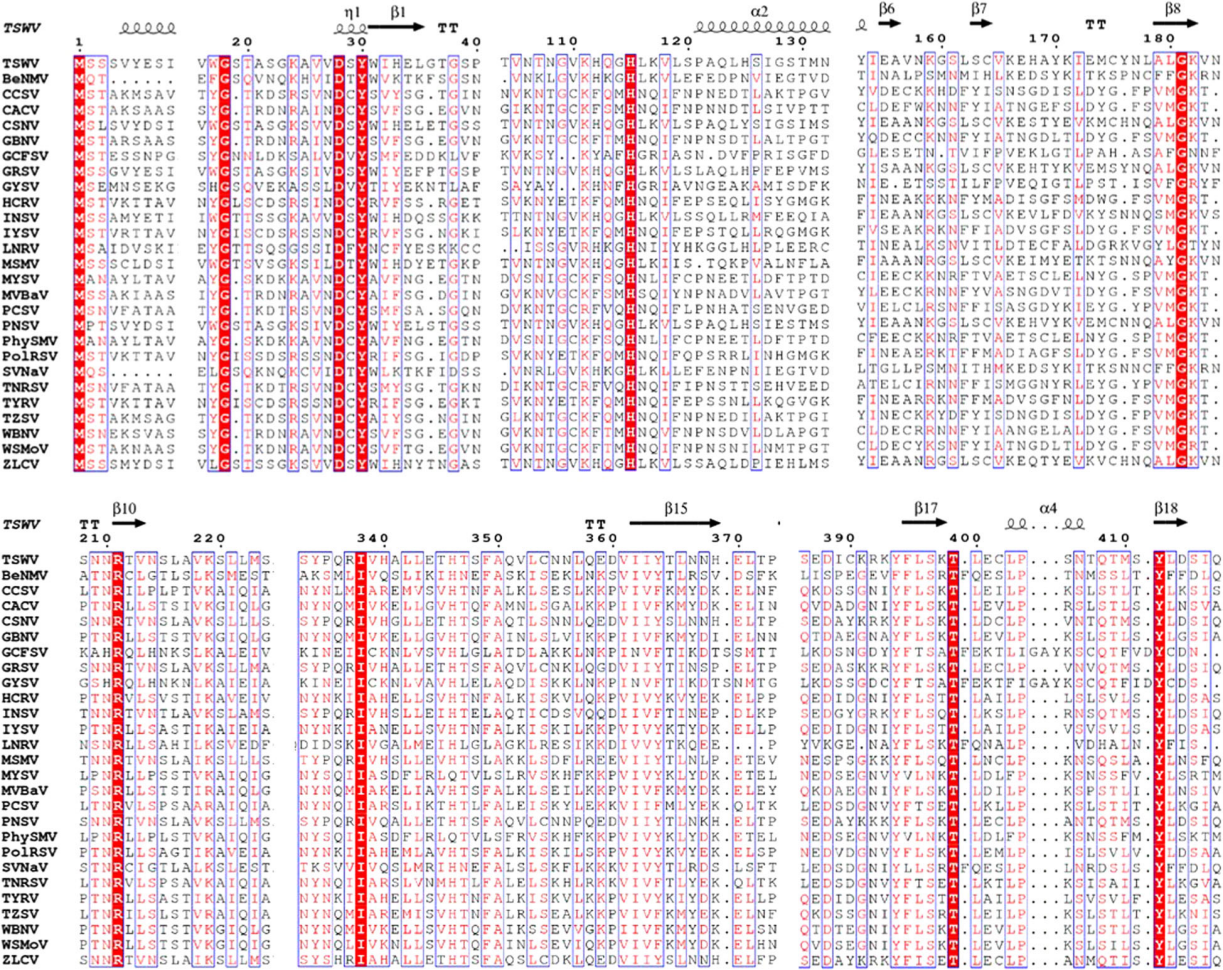
Alignment of the amino acid sequences of the NSs protein of all known tospoviruses. The secondary structure of TSWV predicted by MULTICOM is shown above the alignment with arrows and squiggles indicating beta sheets and alpha helices, respectively. Amino acid residues conserved among all known tospoviruses are highlighted in red. The figure was prepared using ESPript 3.0 server [40]. An extended version can be find in Additional file 3: Figure S2

Sequence comparisons identified nine conserved residues in the N protein, and ten in the NSs protein across all known tospoviruses and are shown in Figs. 3 and 4 (extended versions in Additional file 2: Figure S1 and Additional file 3: Figure S2).

### The N protein model

**Fig. 5:**
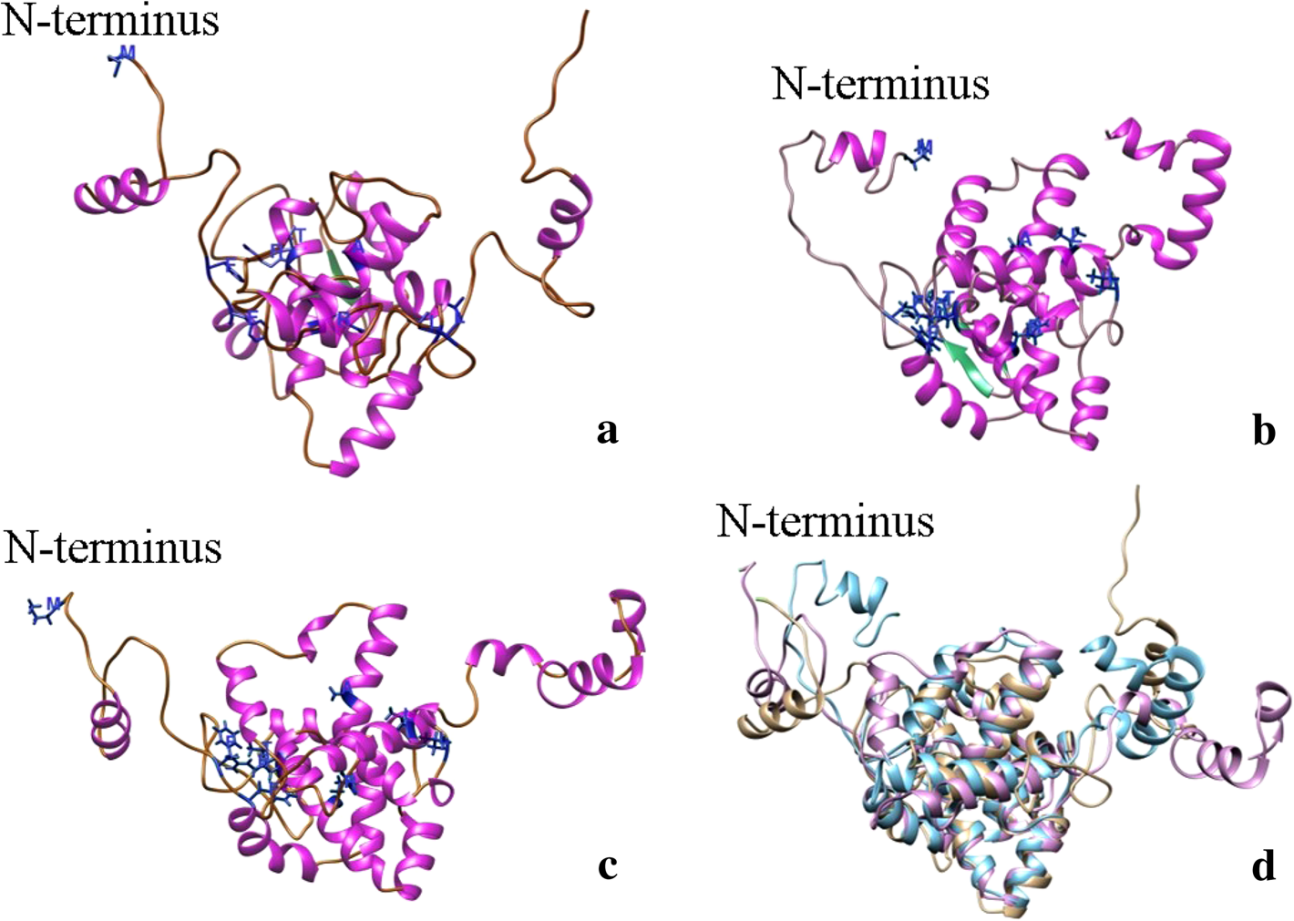
TSWV nucleocapsid protein model based on: **a** MULTICOM; **b** I-TASSER; **c** ROSETTA, top ranked models by consensus score. **d** Superposition of the three models MULTICOM in brown, I-TASSER in blue and ROSETTA in pink. Conserved amino acid residues of all 31 species of the *Tospovirus* genus (M^1^, F^32^, F^34^, T^92^, R^95^, R^101^, L^132^, A^167^ and L^219^) are highlighted in the model

A total of 15 models were predicted by MULTICOM-CLUSTER, MULTICOM-NOVEL and MULTICOM-CONSTRUCT and ranked by the web server APOLLO [56], a quality assessment tool to rank the models to determine the five most representatives. A general assessment tool (QProb) was then used to select the most representative of the five. The MULTICOM-CONSTRUCT model was found to be the most representative of the modeler with a score of 0.496. The N protein model was predicted based on the template Leanyer orthobunyavirus nucleoprotein-ssRNA complex (4J1GA), a protein of 233 amino acids in complex with ssRNA. This model consisted of two β-sheets and 13 α-helix (Fig. 5).

I-TASSER predicted five different models, using crystal structures of the nucleocapsid proteins from Schmallenberg virus (3zl9 and 4jng), Leanyer orthobunyavirus nucleoprotein (4j1j), NheA component of the Nhe toxin from *Bacillus cereus* (4k1p_A), the nucleocapsid protein from *Bunyamwera virus* (3zla), and leoA bacterial dynamin GTPase from ETEC (4aurA) as 10 threading templates. 3ZL9 corresponds to the crystal structure of the nucleocapsid protein from the Schmallenberg virus, an emergent orthobunyavirus in Europe. A similar result was predicted by MULTICOM-CONSTRUCT with the protein 4J1GA as the template. The first model had a C-score of 2.18, an estimated TM-score of 0.46 (±0.15), and an estimated RMSD of 10.9 (±4.6 Å). The Qprob score of the model 3 was 0.55, which represented the best possible common model. This model consisted of two β-sheets and 14 α-helix (Fig. 5).

ROSETTA provided comparative models from structures detected and aligned by HHSEARCH, SPARKS, and Raptor. Five full models were predicted based on the template 4j1jC_309 (Leanyer orthobunyavirus nucleoprotein). All models had the same remark score (0.46) with a confidence score of 0.4629. Qprob score of 0.549 showed ROSETTA Model 5 as one of the best common models. A Qprob score of > 0.5 represents the best possible model by all three models. This model consisted of 17 α-helices and no β-sheets (Fig. 5).

Based on the Qprob analysis, I-TASSER’s Model 3 ranked first with a score of 0.55 while ROSETTA’s Model 5 ranked in second place with almost the same value, 0.549. MULTICOM models ranked 11 to 15, with the Model 3 being the best with 0.496. The models showed two β-sheets, which were consistently located near the amino termini in the positions F^72^ T^73^ F^74^ and I^77^T^78^I^79^. The number of α-helices varied from 12 to 17, and these were distributed throughout the protein. Consistently, all models showed one α-helix near to the amino termini and one to three the carboxyl termini, while the others were in the globular region of the protein (Fig. 5).

**Fig. 6:**
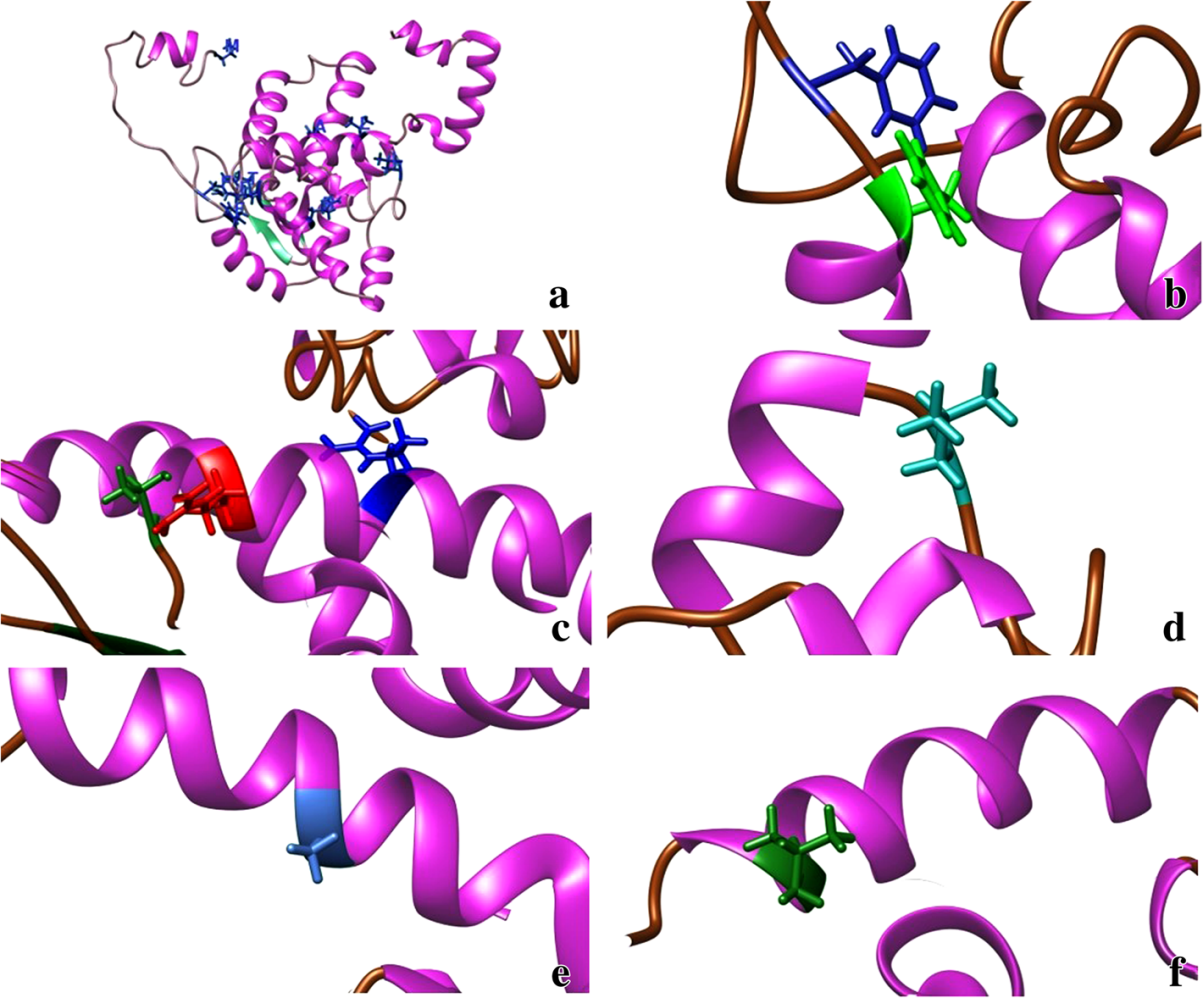
TSWV nucleocapsid protein. Conserved amino acid residues of all 31 species of the *Tospovirus* genus based on the prediction model. **a** I-TASSER model 2; **b** F^32^, F^34^; **c** T^92^, R^95^, R^101^; **d** L^132^; **e** A^167^; **f** L^219^

Nine conserved residues were identified based on the alignment of the N proteins of 31 known orthotospoviral species (Fig. 3). These included M^1^, F^32^, F^34^, T^92^, R^95^, R^101^, L^132^, A^167^ and L^219^ as shown in the models (Figs. 5 and 6). However, if Lisianthus necrotic ringspot virus (LNRV) is excluded from the alignment, the number of conserved amino acid residues has increased to 17, including the nine mentioned above with an additional L^14^, G^147^, G^148^, Q^170^, G^178^, I^179^, T^186^ and P^224^. Some conserved amino acids are in the β-sheets. F^32^, T^92^ and L^132^ were consistently located in the coil in all models, while R^95^, R^101^ and A^167^ were in α-helix in all models. The exceptions were F^34^ and L^219^, which were in the coil in the MULTICOM model, while in I-TASSER and ROSETTA they were found in the α-helix. The structure predicted by ROSETTA was similar to the one by I-TASSER, except that ROSETTA lack the β-sheets and has one additional α-helix near the carboxyl termini (Fig. 5).

The tertiary structure of the globular core was predicted similar by all the algorithms, however there were variations among the three modelers in the C and N arms (i.e., spanning the core globular region of the protein).

### NSs protein

**Fig. 7:**
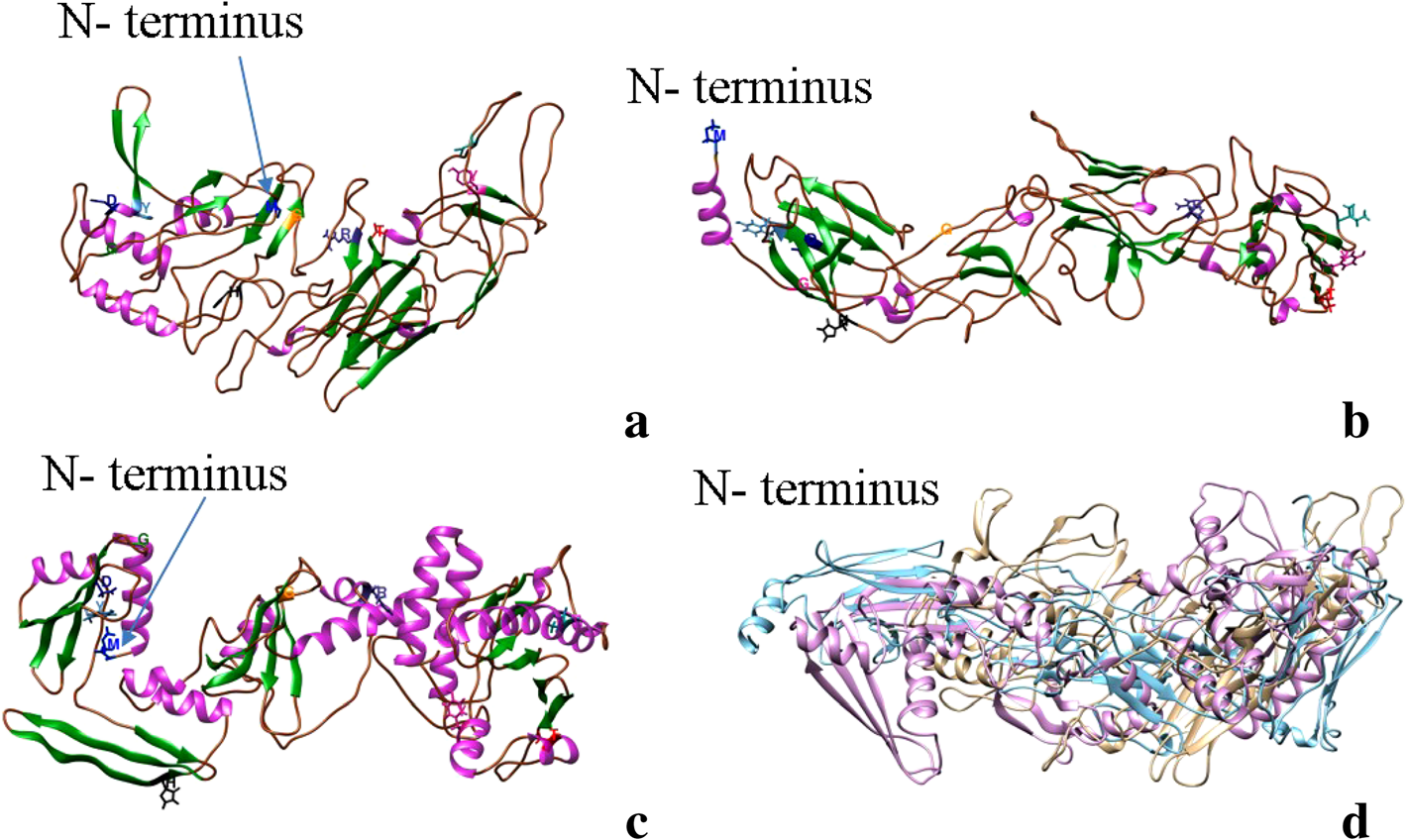
TSWV nonstructural (NSs) protein model based on: **a** MULTICOM-CLUSTER; **b** I-TASSER; **c** ROSETTA modeler, and **d** Superposition of the three models MULTICOM in brown, I-TASSER in blue and ROSETTA in pink. The nine amino acid residues M^1^, G^18^, D^28^, Y^30^, H^115^, G^181^, R^211^, I^338^, T^399^, and Y^412^, conserved in all NSs proteins of the *Tospovirus* genus are highlighted in the TSWV NSs protein model

The models predicted for the N protein were simulated based on other bunyaviral proteins. However, for NSs protein, no bunyavirus-based proteins are available. We use diverse approaches to predict the 3D models as folding structure prediction and residue-contact prediction methods. The MULTICOM, I-TASSER and ROSETTA servers did not find any significant structurally homologous template sequences. Most predicted outcomes had long tail-like regions without a secondary structure because of the unavailability of comparable templates. A total of 15 models were predicted by MULTICOM servers and ranked by APOLLO, while five predictions were made by I-TASSER were ranked based on C-SCORE, and ROSETTA predicted five models (Fig. 7). The models predicted by MULTICOM server were ranked by APOLLO, and Model 1 was ranked first with an average score of 0.161 and a TM score of 0.21. However, Model 4, with an average score of 0.14 and a TM score of 0.189 was the first MULTICOM model ranked by Qprob score with 0.429 (Table 3). This was built based on the template of Phosphonic Arginine Mimetics protein (4K5LA), an inhibitor of the M1 Aminopeptidases from *Plasmodium falciparum*. The MULTICOM model consisted of 23 β-sheets and 5 α-helices.

**Table 3:** Qprob score rank of TSWV non-structural protein, NSs

TSWV NSs Comparison between models	Score
TSWV-NSs-ROSETTA Model 5	0.498
*TSWV-NSs-ROSETTA Model 3*	0.492
TSWV-NSs-ROSETTA Model 1	0.481
TSWV-NSs-ROSETTA Model 2	0.464
TSWV-NSs-ROSETTA Model 4	0.463
TSWV-NSs-I-TASSER Model 5	0.442
*TSWV-NSs-I-TASSER Model 3*	0.435
TSWV-NSs-MULTICOM-Model 4	0.429
TSWV-NSs-MULTICOM-Model 1	0.418
TSWV-NSs-MULTICOM-Model 2	0.418
TSWV-NSs-MULTICOM-Model 3	0.415
TSWV-NSs-I-TASSER Model 2	0.411
*TSWV-NSs-MULTICOM-Model 5*	0.409
TSWV-NSs-I-TASSER Model 1	0.396
TSWV-NSs-I-TASSER Model 4	0.369

For each modeller, the italicized models were chosen for comparison

I-TASSER prediction was built based on combine threading, ab initio modeling and structural refinement approach with the top proteins (3cm9_S), (2gx8 1flg_A), (3txa_A), (2ocw_A) and (1xpq_A). The protein 3CM9 corresponds to a Solution Structure of Human SIgA2 protein, which is the most prevalent human antibody and is central to mucosal immunity. However, predictions from all the servers had a low C-SCORE due to the lower identity with the templates. The Model 5 was selected based on a Qprob score of 0.442 (Table 3). This model consisted of 12 β-sheets and 2 α-helices.

ROSETTA’s prediction used a fragment assembly approach, and the predicted models were based on the following templates: *Tetrahymena thermophila* 60S ribosomal subunit in complex with initiation factor 6 (4V8P), the chaperone human alpha-crystallin domain (2y22A_301), the crystal structure of ARC4 from human Tankyrase 2 (3twqA_201), and the binding domain of Botulinum neurotoxin DC in complex with human synaptotagmin I (4isqB_101) and Lipid-induced Conformational Switch Controls Fusion Activity of Longin Domain SNARE Ykt6 (3KYQ). The Model 5 of ROSETTA was the top ranked model by Qprob score of 0.498 and consisted of 17 β-sheets and 18 α-helices.

However, for this protein, we found no agreement between the 3D models generated by the three methods. The average pairwise TM-score of 0.18 and RMSD of 31.1 Å among the top models predicted by each method, showed random structural similarity between the predictions from the three servers, making the predicted models unreliable to interpret or assign any biological significance.

Based on the single model quality assessment tool Qprob, ROSETTA’s Model 5 ranked first with a score of 0.498, while I-TASSER’s Model 5 ranked in 6th place with 0.442 and the MULTICOM’s Model 4 ranked 8 with a 0.418 score.

Despite the complexity of the protein and the lack of crystalized templates, we used another strategy to obtain a better prediction of 3D model of the NSs protein. The NSs protein sequence was divided into two fragments (domains) with the software DoBo and used the two sequences to predict structures for Domain-I of 254 residues at the amino termini, and domain-II of 213 residues at the carboxyl termini. Then, using CONFOLD, we predicted new models based on a residue-contact method of the two domains and obtained five 3D models for each domain.

The Model 1 of the Domain 1 consisted on three β-sheets and five α-helices, while the Model 1 of the Domain 2 showed two β-sheets and seven α-helices. In total, both domains showed evidence of five β-sheets and 12 α-helices for the NSs protein. In comparison, the residue-contact method predicted fewer number of β-sheets and α-helices than the ab initio methods.

Ten conserved residues were identified based on the alignment of 27 sequences of different tospoviral species. Using TSWV as the reference sequence, the conserved residues are M^1^, G^18^, D^28^, Y^30^, H^115^, G^181^, R^211^, I^338^, T^399^, and Y^412^ were highlighted in the models (Fig. 7). Because there is no similarity among the models predicted, the localization of the conserved residues was variable among them. Only M^1^ and G^18^ were located in a coil region in the four predictions, while D^28^ and Y^30^ were in an α-helix by MULTICOM prediction, in a β-sheet in I-TASSER and ROSETTA, but in a coil region in the Domain 1 in the CONFOLD model. H^115^ was in a coil region by MULTICOM, in α-helix by I-TASSER and CONFOLD, but in β-sheet by ROSETTA. G^181^ where located in β-sheet by two modelers and in a coil region in the other two. I^338^and T^399^ were in a coil region in MULTICOM and I-TASSER, while in ROSETTA and CONFOLD domain 2 were located in a α-helix. R^211^ and Y^412^ was inconsistently located in either coil, β-sheet or α-helix through the four predictions.

## Discussion

**Fig. 8:**
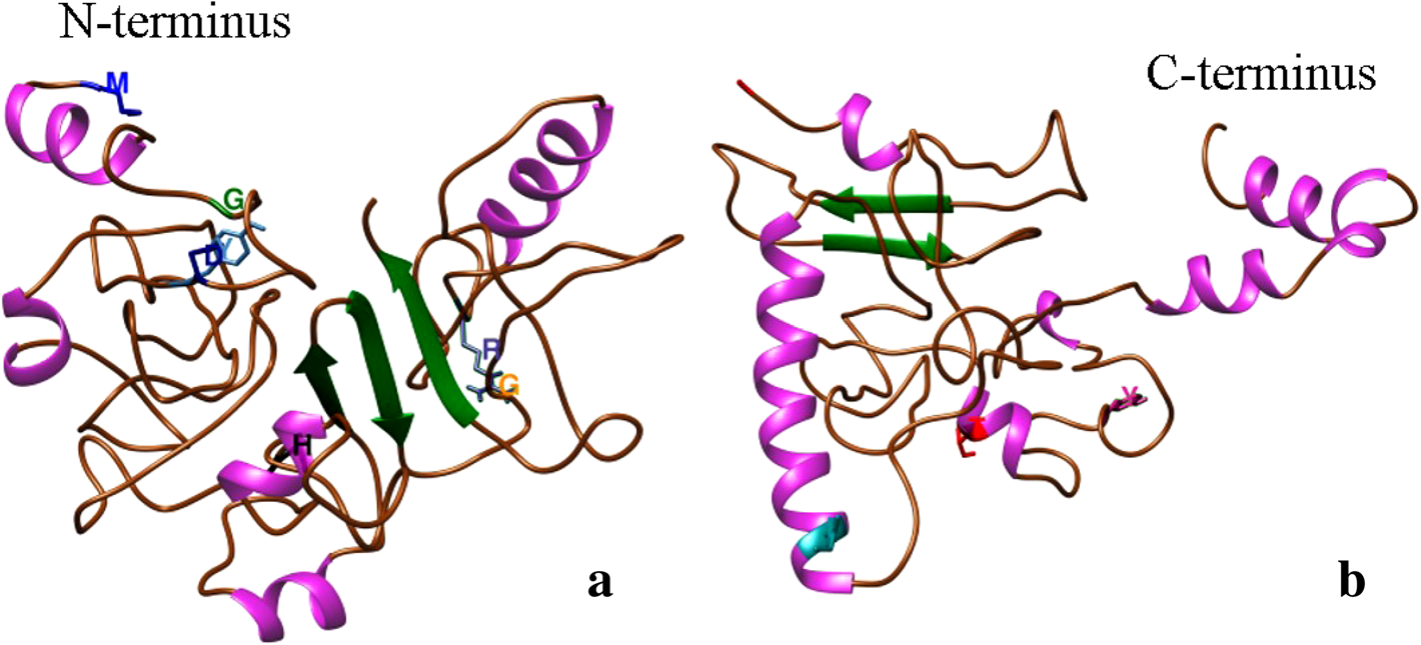
TSWV nonstructural (NSs) protein prediction model based on residue-contact method, CONFOLD: **a** Domain 1; **b** Domain 2

In this study, we first identified family-wide conserved amino acid residues, and then used three distinct programs to first predict the 3D structures of N and NSs proteins, and one additional program (CONFOLD) for the NSs protein only (Fig. 8), followed by their potential localization. While the structure of N proteins is available for some members of the order *Bunyavirales*, no such information is available for NSs. We used N protein as our reference to verify the accuracy of prediction by the three modelers before using them to predict the NSs structure. Both proteins play important roles in viral infection, pathogenesis and assembly. The prediction models of the tospoviral protein structures are an attempt to provide a new understanding of the viral structure.

Among the members of *Bunyavirales*, the N protein structure of the orthobunyaviruses La Crosse orthobunyavirus (LaCV) [33], *Bunyamwera* virus (BUNV) [31], Schmallenberg virus (SBV) [32], Leanyer virus (LEAV) [70], the Nairovirus *Crimean-Congo hemorrhagic fever virus* (CCHFV) [71] and the Phlebovirus *Rift Valley fever virus* (RVFV) [72] were determined by crystallization. Among tospoviral proteins, the glycoproteins [34] and the N protein of TSWV and GRSV have been predicted by folding prediction [8, 35], but so far only N protein structure of TSWV has been determined by crystallization [36–38].

Soundararajan et al. [34] reported a theoretical model of TSWV glycoprotein (G_N_/G_C_) using I-TASSER, and obtained a model folding of G_N_ and G_C_ with a C-SCORE of − 2.73 and − 0.93 respectively. It was concluded that the structural organization of the envelope glycoprotein could be the primary factor to cause the G_C_ arrest in ER. Also, their protein-protein interaction study indicated that the C-terminal region of G_N_ is necessary for the Golgi retention and dimerization of the G_N_ to the G_C_.

Komoda et al. [36, 37] crystallized the bacterially expressed TSWV N protein. Li et al. [8] built a three-dimensional homology model of TSWV N protein using I-TASSER. The model was composed of N-arm, N-terminal domain, C-terminal domain, and C-arm, where the N- and C-terminal domains formed a core structure. Their data suggested that amino acids R^94^/ R^95^ and K^183^/Y^184^ are important for N binding to RNA and those amino acids were mapped onto a charged surface cleft of the three-dimensional structure of the N homology model. In our study, R^95^ was conserved among all 31 species of the genus *Tospovirus* and was consistently located in a α-helix by all three models in agreement with the structures reported by Komoda et al. [37] and Guo et al. [38]. Interestingly, Guo et al. [38] found in their crystallized structure, that R^95^ is important for the protein folding and RNA binding.

In our study, we used the three most popular modelers available: I-TASSER, MULTICOM and ROSETTA for predicting the tertiary structures. All three modelers use different approaches for model building and thus each of them selected a different bunyavirus N protein as a template. The folding pattern obtained for the three models was similar to each other, and they consisted of a globular core shape containing two β-sheets and 12 to 17 α-helix, and two terminal chains corresponding to the N and the C termini exposed on the surface of the protein. Visually, our predictions agreed with those by Li et al. [8]. Additionally, by using a superimposed match maker, we found agreement between our first score I-TASSER model with those from Komoda et al. [37] and Guo et al. [38] (Additional file 4: Figure S3). The main groove region shared similar structure, however there are folding differences in the N- and C-termini in all three models. The predictions by Komoda et al. [37] and Guo et al. [38] differed from each other in the number of beta-sheets and alpha helix, while Komoda et al. presented 4 and 12, and Guo et al. showed 2 and 13, respectively. Our I-TASSER prediction, β-sheets located in residues F^72^T^73^F^74^ and I^77^T^78^I^79^ corresponded with those from Guo et al., and the β-sheets #2 and #4 from Komoda et al. As Guo et al. state, their structure was most in agreement with that of Komoda et al., with some differences in the arms. Both structures were determined based on polymeric crystals, building an asymmetric ring of three protomers. When the single protomers were extracted from the multimeric PDB files to compare with our prediction, Komoda’s structure had extra residues of 21 amino acids from the expression vector at the N-terminus, while Guo’s structure lacked some residues: two residues (M^1^ and S^2^) at the N-terminus, and residues K^19^ to E^25^ in the N-arm. Additionally, both structures present an Alanine mutation in residue T^255^ to give stability to the crystal. This variation can be different from one protomer to another in the same trimeric structures. The superimposed model of the Chain A from Komoda et al. [37] and Guo et al. [38] prediction allowed us to visualize these differences, but also can help to explain the variation in the N-arm from all the models (Additional file 4: Figure S3). Our predicted model, based on threading approach randomly selected the most similar models, when the crystal structures for TSWV N were not available. Luckily, having these structures recently made available in the database, allowed us to test the accuracy of our models. This coincidence helped us to have more confidence in the models predicted using similar approaches for the NSs protein.

Initially, we used the same approach to predict the 3D structure of TSWV NSs protein. However, there was no similar protein crystallized from any virus in the order *Bunyavirales*. All the modelers selected different templates and approaches to predict. In this case, only the prediction by ROSETTA was different to the one by I-TASSER and MULTICOM. The NSs protein, a suppressor of host plant’s defense, is a member of the pfam03231 Bunya-NS-S2 protein family and had been shown to interfere with host (animal, human and plants) defense response. It is interesting that I-TASSER used the protein 3CM9, which is central to the human mucosal immunity, as one of the templates for NSs in the combined threading prediction (Fig. 7).

The top models predicted by each method, showed no similarity between the predictions from the three servers, making the predicted models unreliable to assign any biological significance. Hence, we resorted to other options to predict the structure for the NSs protein and used contact-guided structure prediction to build 3D models making use of predicted residue-contacts.

3D models of silencing suppressor proteins bound to siRNA based on crystal structure are available for plant viruses, such as p19 of *Carnation Italian ringspot virus* (CIRV) [39]; p19 of *Tomato bushy stunt virus* (TBSV) [40]; and p2b of *Tomato aspermy virus* (TAV) [41]. The p21 of *Beet yellows virus* (BYV-*Closterovirus*) was crystallized and binding domains determined [73]. However, for other viruses the silencing suppressor protein has not been crystallized yet, and hence in silico prediction was used to determine their structure. Costa et al. [74] found that p23, one of the three silencing suppressor proteins of *Citrus tristeza virus* (CTV), was able to transiently suppress the local but not the short-range silencing. They predicted a 3D model structure of the p23 protein using I-TASSER modeler, which showed differences within the Zn-finger region, between isolates. As the p23 has not been crystallized yet, the prediction helped to support the functional studies of the protein.

de Ronde et al. [19] found in TSWV that a single amino-acid mutation in G*W*/WG motif (position 17/18) resulted in dysfunctionality of NSs for RSS and Avr activity suggesting a putative interaction with Argonaute 1 (AGO1). Hedil et al. [14] confirmed W17A/G18A residues may play an important role in the ability of NSs to interfere in the RNA silencing pathway further downstream siRNA biogenesis and sequestration. G^18^ in TSWV was conserved among all 27 species of the genus *Tospovirus* and was the only amino acid consistently located in a coil region in all four methods used to predict the NSs 3D model. Zhai et al. [21] found that the residues K^182^ and L^413^ in the motifs, GKV/T (181–183) and YL (412–413), in the NSs protein are essential for the protein’s suppressor activity. Based on our study G^181^ and Y^412^ were conserved across the family, but their location in the tertiary structure were not consistent in either a coil, α-helix or β-sheets.

In case of *Watermelon silver mottle virus* (WSMV), Huang et al. [20] showed that mutations at H^113^ in the common epitope (CE) (^109^KFTMHNQ^117^) and Y^398^ at the C-terminal β-sheet motif (^397^IYFL^400^) affect NSs mRNA stability, and protein stability, respectively, and concluded that both are critical for silencing suppressor activity of NSs. The H^113^ of WSMV corresponds to H^115^ in TSWV sequence and is also conserved in all species of the genus. This amino acid was in the coil region in three of the models and in a β-sheet in the ROSETTA model. The fact that selected residues identified in this study were conserved across the *Tospovirus* genus suggests that they could be functionally critical for the N and NSs proteins. These regions in the N and NSs genes thus could be potential targets for novel virus suppression strategies.

Considering the limitations on structural folding of a large (NSs) protein, and due to the low scores, at this point of time, we cannot say with a high degree of confidence that the predictions for the NSs protein are not random. Our efforts to verify and/or validate the prediction has been hampered by the fact that there are no NSs proteins structures determined by crystallization for any known tospoviruses or members of the order *Bunyavirales* that we could use for comparison. Furthermore, we are constrained by the fact that the known proteins with silencing suppressor activity of other viruses didn’t share any folding homology that we can use as a template or to validate our models.

Juxtaposition of the conserved residues could provide us with insights into potential interactions among the residues. In case of the NSs protein, there was no consistent pattern with respect to co-localization of the conserved residues. The inter- and intra-interactions between and among the various conserved residues should be discerned to determine the stability of the protein and the possible residues involved in the functions of the protein either in silico or in vitro analysis. While Li et al. [8] used I-TASSER for the prediction folding of N protein, we used two additional independent modelers, ROSETTA and MULTICOM to enhance the stringency of the predictions. CONFOLD could generate models comparable to those generated by other state-of-the-art tools such as ROSETTA and FRAGFOLD. However, due to the lack of an accurate template, CONFOLD could not be used to generate a non-random model. Because there are currently no structural homologs available that could be used for homology modeling, the results produced by different modeling platforms were not congruent and validation await the availability of crystallization data for NSs. While it is important to evaluate the stereochemical quality of the obtained structural models and to compare it to that of the X-ray structures which were used as a template, again this effort was hampered by lack of a ‘good’ template hit. The availability of an infectious clone would facilitate reverse genetics to test, verify, and validate the potential role(s) of some of these conserved residues with respect to their relative location in the tertiary form of the protein. However, a reverse genetics system is not yet available for any tospovirus. 3D model prediction can be a valuable tool when there are limitations in the biological order such as the absence of a reverse genetics system or the lack of crystalized structures, nearly homolog to the query.

The residues identified in the N protein, M^1^, F^32^, F^34^, T^92^, R^95^, R^101^, L^132^, A^167^ and L^219^, and in the NSs protein, M^1^, G^18^, D^28^, Y^30^, H^115^, G^181^, R^211^, I^338^, T^399^, and Y^412^, are genus-wide conserved and some of them are already known to play critical roles in the proteins functions. The mRNA sites for residues, for example, R^95^, in N protein can be used as a target by RNAi approach and the residues identified in the amino and carboxy termini of the N protein, can potentially be targeted at the protein level.

This is the first report to localize genus-wide conserved residues in N and NSs proteins and determine the structural features of the NSs of any tospovirus through folding and residue-contact prediction methods. Determining a reliable protein structure will lead to the identification of critical regions that could be susceptible to targeted approaches for novel viral control methods. Molecular dynamics studies need to be done for a better understanding of the interactions among the various models.

## Conclusion

Predicted 3D structures of tospoviral NSs protein allowed to find a consistent location for two of the nine conserved residues among all members of the genus *Tospovirus*. The modelers used gave accurate prediction for N protein allowing the localization of the conserved residues. Our results form the basis for further work on the structure-function relationships of tospoviral proteins and could be useful in developing novel virus control strategies targeting the localized residues.

## Additional files

Additional file 1: **Table S1.** List of programs used to process the amino acid sequences and predict the models. (DOCX 13 kb)

Additional file 2: **Figure S1.** Extended version of the alignment of the nucleocapsid amino acid sequences of all known tospoviruses. The list of tospoviruses used is given in Table 1. The columns highlighted in red indicate amino acid residues conserved among all known tospoviruses. The secondary structure of TSWV predicted by I-TASSER is shown above the alignment with arrows and squiggles indicating beta sheets and alpha helices, respectively. Amino acid residues conserved among all known tospoviruses are indicated in red. The figure was prepared using ESPript 3.0 server [40]. (PDF 29 kb)

Additional file 3: **Figure S2.** Extended version of the alignment of NSs amino acid sequences of the NSs protein of all known tospoviruses. The secondary structure of TSWV predicted by MULTICOM is shown above the alignment with arrows and squiggles indicating beta sheets and alpha helices, respectively. Amino acid residues conserved among all known tospoviruses are highlighted in red. The figure was prepared using ESPript 3.0 server [40]. (PDF 34 kb)

Additional file 4: **Figure S3.** Superimposed model of TSWV N protein crystal structure from Komoda et al. [37] -5iP1 chain A- (brown color), Guo et al. [38] -5y6j chain A- (blue color) and I-TASSER prediction model (This study) (purple color). A) front view, B) rotate 180° horizontal, C) rotated 90° vertical (upper view), D) rotated 270° vertical (bottom view). (JPG 240 kb)

## Abbreviations

ANSV: Alstroemeria necrotic streak virus
Avr: Avirulence
BeNMV: Bean necrotic mosaic virus
CaCV: Capsicum chlorosis virus
CASP: Critical Assessment of Protein Structure Prediction
CCSV: Calla lily chlorotic spot virus
CSNV: Chrysanthemum stem necrosis virus
GBNV: *Groundnut bud necrosis virus*
G_C_: Glycoprotein carboxy
GCFSV: *Groundnut chlorotic fan- spot virus*
GDT-TS: Global-distance test
G_N_: Glycoprotein amino
GRSV: *Groundnut ringspot virus*
GYSV: *Groundnut yellow spot virus*
HCRV: Hippeastrum chlorotic ringspot virus
INSV: *Impatiens necrotic spot virus*
IYSV: *Iris yellow spot virus*
LNRV: Lisianthus necrotic ringspot virus
MSMV: Melon severe mosaic virus
MVBaV: Mulberry vein banding associated virus
MYSV: Melon yellow spot virus
N: Nucleocapsid
NSs: Silencing suppressor gene
PCSV: Pepper chlorotic spot virus
PhySMV: Physalis silver mottle virus
PNSV: Pepper necrotic spot virus
PolRSV: *Polygonum ringspot virus*
RdRp: RNA-dependent RNA-polymerase
RMSD: Root mean square deviation
ssRNA: Single stranded RNA
SVNaV: Soybean vein necrosis associated virus
TCSV: Tomato chlorotic spot virus
TM-score: Template modelling score
TNeV: Tomato necrosis virus
TNRV: Tomato necrotic ringspot virus
TNSV: Tomato necrotic spot virus
TSWV: *Tomato spotted wilt virus*
TYRV: Tomato yellow ring virus
TZSV: Tomato zonate spot virus
WBNV: *Watermelon bud necrosis virus*
WSMoV: *Watermelon silver mottle virus*
ZLCV: *Zucchini lethal chlorosis virus*
