# Supplementary material for: Identification and localization of Tospovirus genus-wide conserved residues in 3D models of the nucleocapsid and the silencing suppressor proteins
Source: Virol J. 2019 Jan 11;16:7. doi: 10.1186/s12985-018-1106-4 (PMC6330412; doi:10.1186/s12985-018-1106-4)
Supplement: Supplementary file 1 — Table S1. List of programs used to process the amino acid sequences and predict the models. (DOCX 13 kb) [file 12985_2018_1106_MOESM1_ESM.docx]

**Additional File 4: Table S1. List of programs used to process the amino acid sequences and predict the models.**

| Software | Reference |
| --- | --- |
| MEGA 6.06 | 42 |
| ESPript 3.0 server | 43 |
| ROSETTA | 44 |
| I-TASSER | 45, 46,47 |
| MULTICOM-CONSTRUCT | 48 |
| MULTICOM- CLUSTER | 48 |
| MULTICOM- NOVEL | 50 |
| APOLLO | 56 |
| Q-score | 58 |
| UCSF Chimera version 1.10.1 | 60 |
| DoBo | 66 |
| ThreaDom | 65 |
| MetaPSICOV | 64 |
| JackHammer | 67 |
| PSIPRED | 68 |
| BETApro | 41 |
| CONFOLD | 69 |
