# Supplementary material for: Identification and localization of Tospovirus genus-wide conserved residues in 3D models of the nucleocapsid and the silencing suppressor proteins
Source: Virol J. 2019 Jan 11;16:7. doi: 10.1186/s12985-018-1106-4 (PMC6330412; doi:10.1186/s12985-018-1106-4)
Supplement: Supplementary file 2 — Figure S1. Extended version of the alignment of the nucleocapsid amino acid sequences of all known tospoviruses. The list of tospoviruses used is given in Table 1. The columns highlighted in red indicate amino acid residues conserved among all known tospoviruses. The secondary structure of TSWV predicted by I-TASSER is shown above the alignment with arrows and squiggles indicating beta sheets and alpha helices, respectively. Amino acid residues conserved among all known tospoviruses are indicated in red. The figure was prepared using ESPript 3.0 server [40]. (PDF 29 kb) [file 12985_2018_1106_MOESM2_ESM.pdf]

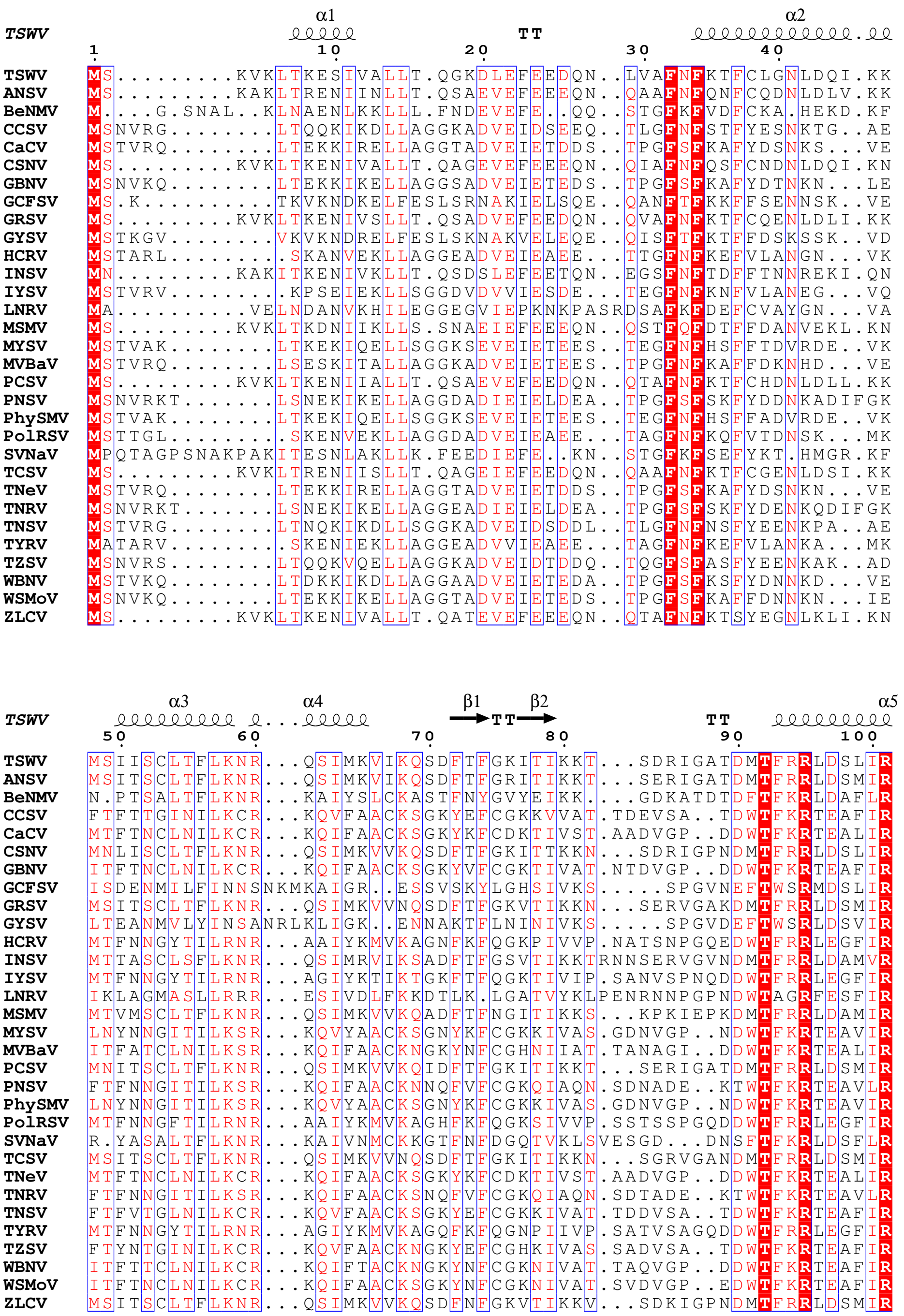
TSWV
α1
α2
α3
α4
β1
β2
α5
TT
TSWV
ANSV
BeNMV
CCSV
CaCV
CSNV
GBNV
GCFSV
GRSV
GYSV
HCRV
INSV
IYSV
LNRV
MSMV
MYSV
MVBaV
PCSV
PNSV
PhySMV
PolRSV
SVNaV
TCSV
TNeV
TNRV
TNSV
TYRV
TZSV
WBNV
WSMoV
ZLCV

```
                                   α6                  α7                   α8
TSWV
                          110              120              130              140            150
TSWV     VRLVEETG.....NSENL..NTIKSKIASHPLIQAYGLTLD...DAKSVRLAIMLGGGLP
ANSV     VKLIQETA.....NAVNL..DAIKTKIASHPLVQAYGLPLN...DAKSVRLAIMLGGSIP
BeNMV    VKLLTHCTKIWDGTDPNAQ.TALCSELAKIPIVKAYGLEVS...DKQKCYIAIVLGGNLS
CCSV     AKLISMVEHTTDEATKKL....MYTKAMELPLVVAYGLNVPAEFHYAALRLMLCIGGPLP
CaCV     VKMINMIEKNKNEAAKQE....MYSKVMELPLVAAYGLNIPASFDSCALRMMLCIGGPLP
CSNV     VKLIGRTK.....SDEDL..NTIKSKIASHPLVQAYGLSLN...DAKSVRLAIMLGGSLP
GBNV     TKMASMVEKSKNDAAKQE....MYNKIMELPLVAAYGLNVPASFDTCALRMMLCIGGPLP
GCFSV    MKYIERVKDYGDDRMKAEN.....AKLTNWLLEVFN.LHTDAMSPRDEVIFKVVTGGNLN
GRSV     VKLIEETA.....NNENL..AIIKAKIASHPLVQAYGLPLA...DAKSVRLAIMLGGSIP
GYSV     MKYIDRIKDYNEDKLKAES.....AKLNNWLLEIFN.LK..QMSPKDEVIFKVITGGNLN
HCRV     AKMFMELLAVENSTEQQK....MYEKLCDLPMVNAYGLKPSPRFDATTARVMLTLGGPLP
INSV     VHLVGMIKD....NGSALT.EAINS.LPSHPLIASYGLATT...DLKSCVLGVLLGGSLP
IYSV     ARMLVELIETKDEKEKQK....MYEKICGLPLVSAYGLKPSSKFHATTARIMLTLGGPLT
LNRV     YR.ICLKAESDKELAKT.....SIEILQDTPLAYIYGLKSMPINDTDDMKLIACFSLRLN
MSMV     AKMIEFTA.....KDEAL..EIIKVKISSHPLVMAYSLDVN...DAKSARIACLLGGSLP
MYSV     TLMISIAEKTENEEEKQK....MYEKAMQLPLVAAYGLTVPAKFDMTALRLMLCIGGPLS
MVBaV    VKMIRMVEK....AASNESKLAMYQKVMELPLVSAYGLNAPPEFNACALRLMLCIGGPLS
PCSV     VKLIEETA.....NAENL..LAIKTKIASHPLVQAYGLPLT...DAKSVRLAIMLGGSIP
PNSV     VLMSKMVEECTTQKLQQD....MYTKLMSLPMVIAYGLNVPPNFDVAATRLMLCVGGPLP
PhySMV   TLMISIAEKTENEEEKQK....MYEKAMQLPLVAAYGLTVPAKFDMTALRLMLCIGGPLS
PolRSV   AKMFMELINMENAVEQQK....MYEKLCELPMVNAYGLKPSSKFDPTTARVVLTLGGPLP
SVNaV    VKMLEHNFAVFDGTNEEAK.QSLCNDLATIPLVQAYGLTVK...DKMSAKLAIMIGGSLP
TCSV     VKLIEETG.....KAENL..AIIKSKIASHPLVQAYGLPLT...DAKSVRLAIMLGGSIP
TNeV     VKMINMIEKNKNEAAKQE....MYSKVMELPLVAAYGLNIPASFDSCALRMMLCIGGPLP
TNRV     VLMAKMVEECTTQKLQQD....MYSKLMSLPMVIAYGLNVPPNFDSCATRLMLCVGGPLP
TNSV     AKLISMIEKTGDETSKKQ....MYTKVMELPLVSAYGLNVPNEFSSAALRLMLCIGGPLP
TYRV     AKMFMELIAVENASEQQK....MYEKLCELPMVNAYGLKPSPKFDATTARVMLTLGGPLP
TZSV     TRLISMAEHATDETTKKQ....MYIKAMELPLVAAYGLNVPVDFNSSAIRLMLCIGGPLP
WBNV     TKMVSMVEKSTNENAKQE....MYTKIMELPLVAAYGLNVPAAYDSCALRMMLCIGGPLP
WSMoV    TKIVSMVEKSKNEAARQE....MYGKIMELPLVAAYGLNVPSSYDSCALRMMLCIGGPLP
ZLCV     VKPIEATA.....NDENL..SAIRSKIASHPLVQAYGLSLT...NAKSVRLAIMLGGSIP
```

```
           η1   α9          α10                              α11
TSWV
                  160          170          180          190          200
TSWV     LIASVDSFEMISVVLAIYQDAKYKDLGIDPKKYDTREAL......GKVCTVLKSKAFEMT
ANSV     LIASVDSFEMISVVLAIYQDAKCKDLGINPKKYDTKEAL......GKVCTVLKSKGFSMD
BeNMV    LLASLPGCEVACFALAIFQDLKKNELGIKSD.FDTKDQA......GRVAAVLDAKNFVFG
CCSV     LLSSLPGLAPVCFPLAYFQNVKKEQLGIKN..FSTYEQI......CKVAKVLSAASVEFA
CaCV     LLSSIKGLAPIIFPLAYYQNVKKEKLGIKN..FSTYEQV......CKVAKVLSASQVEFK
CSNV     LIASVESFEMISVVLAIYQDSKHKELGIDEKKYDTKEAL......GKVCTVLKSKGFAID
GBNV     LLSSMTGLAPIIFPLAYYQNVKKEKLGIKN..FSTYEQV......CKVAKVLSASQIEFK
GCFSV    HLMGFKTTFAHAFAIANYQHKRSEELGIKN..FDTKAQLDRMVVIGERCSLLPSP.....
GRSV     LIASVDSFEMISVVLAIYQDAKYKELGIEPTKYNTKEAL......GKVCTVLKSKGFTMD
GYSV     HLMCFKTTFAHAFAIANYQHKRAEQLGITN..FDTKAQLDRMIVIGERNKVLPST.....
HCRV     LMASLDKFAAAAFPLAYFQNVKKEAQGINK..FSTYEQL......CKIARVMATKEFVFT
INSV     LIASVLNFEIAALVPAIYQDAKHVELGIDMSKFSTKEAV......GKVCTVLKSKGYSMN
IYSV     LMASLDIFAAAALPLAYFQNVKKEALGISR..FSTYEQL......CKVARVMAAKEFKFT
LNRV     HLASMPKTCRHAMAVAIYRYHCKDMV...RKEFHDTETLER.....VCTVAKATGFVFD
MSMV     LLASIPHYEAICLVLAVYQDAKSQELGIDQKKYDTKEAI......GKVCTVLKSKGYVMD
MYSV     LLASLHSLCPVVLPLAYFQNVKKEQLGIKN..FSTYEQI......CKIARVMSASNMTFK
MVBaV    LLSSFKALAPIVFPLAYYQNVKKEKLGIKN..FSTYEQV......CKVAKVLSAAQFDFQ
PCSV     LIASVDSFEMISIVLAIYQDAKCKDLGIDPKKYDTKEAI......GKVCTVLKSKGFLMD
PNSV     LLSSLAPMAPITFPLAYFQNVKKQNLGIKN..FSTYEQL......CKVARVCDAAQIEFK
PhySMV   LLASLHSLCPVVLPLAYFQNVKKEQLGIKN..FSTYEQI......CKIARVMSASNMTFK
PolRSV   LMASLDKFAAAAFPLAYFQNVKKEALGINK..FSTYEQL......CKIARVMATKEFTFT
SVNaV    LLASITGCEAYCFGLAIFQDLKKEQLGIVN..FDTKAQA......AKVASVLDAKGFKFT
TCSV     LIASVDSFEMISIILAIYQDAKYKDLGIEPSKYNTKEAL......GKVCTVLKSKGFTMD
TNeV     LLSSIKGLAPIIFPLAYYQNVKKEKLGIKN..FSTYEQV......CKVAKVLSASQVEFK
TNRV     LLSSLPAMAPIAFSLAYFQNVKKQNLGIKN..FSTYEQL......CKVARVCDAAQVEFT
TNSV     LLSSIPGLAPVCFPLAYFQNVKKEQLGIKN..FSTYEQV......CKIAKVLSASGVEFS
TYRV     LMASLDKFAATAFPLAYFQNVKKEALGISK..FSTYDQL......GKIARVMATKEFTFT
TZSV     LLSSVPGLAPVCFPLAYFQNVKKEQLGIKN..FSTYEQI......CKVAKVLSAASVEFT
WBNV     LLSSLTGLAPVIFPLAYYQNVKKEKLGIKN..FSTYEQV......CKVAKVLSASQIEFK
WSMoV    LLSSIRGLAPIIFPLAYYQNIKKEKLGIKN..FSTYEQV......CKVAKVLSASQVEFK
ZLCV     LIASVDSFEMISVVLAIYQDSKHKELGIDLKKYDTTEAL......GKVCSVLRSKGFDID
```

```
TSWV      α12                                η2     α13
          210              220               230              240

TSWV      EDQVKKGKEYAAILSSSN.....PNAKGSVAME..HYSETLNKFYEMFGV.........
ANSV      DTQVTKGREYAAILGSCN.....PNAKGSVAME..HYSEHLNKFYEMFGV.........
BeNMV     EPENEKLKKIAGILKEMT.....PTRRGHAALT..KYAEQLSIISGVIGVTFEMPG....
CCSV      DKTKELFDSTVKLLGDSS.....PGTAGAISLH..KYNSQLKQMEAAFNAKLNVDDFGQN
CaCV      EDLDKLFKQAVKLLSESN.....PGTASSISLK..KYDEQVKYMDKVFSANLSVDDYGEH
CSNV      EQQMEKGKEYANILKACD.....PRMKGAIAME..HYSDSLNKFYEMFGV.........
GBNV      NELEEMFKSAVKLLSESN.....PGTASSISLK..KYDEQVKYMDKAFSASLSMDDYGEH
GCFSV     .........TPKTLIESYFRNAVPKAKSDSKDQSSKYNDLQNAIAAVNFEVPWRQVMLYQ
GRSV      DAQINKGKEYAKILSSCN.....PNAKGSIAMD..YYSDNLDKFYESVGV.........
GYSV      .........APLTLINQYFKNAMPKVKAAERDQSSKYEELQAAIG..NGEL.........
HCRV      GTAKEIFEETIKILNDCT.....PGTTGAASLN..KFNEQIKALENVFG..KIVDDNEAS
INSV      SVEIGKAKQYADILKACS.....PKAKGLAAMD..HYKEGLTSIYSMFNAT......I..
IYSV      EKYKKIFDETIKILTDCT.....PGTSGAASLI..KFNEQIKILEGAFG..KIVEDIG.E
LNRV      ED......FYRIVLKFRN.....AKIISTGAQAVMEYHGSIKKAIESLGGTFNYTPSHRT
MSMV      NDQLEKAKMYAQILSKCD.....PKLKGDMAMN..HYEAGLKQIYEIFGVS......G..
MYSV      KEFDELFKSCVKILADCK.....PGTTSGISLK..IYNEQVQFMEQAFKSSLVVDGMGES
MVBaV     GDFKTLFEEAVKMLSESN.....PGTASSISLK..KYDEQVKYMDKVFSSSLVVDDYGEN
PCSV      ESQVNKGKEYATILNSCN.....PNAKGSVAME..YYSDNLNKFYEMFGV.........
PNSV      GDVEKLFNSATDVLKSST.....PGTASSVSLK..KYSEQISYLEKAFSAKAVVDDFGSN
PhySMV    KEFDELFKSCVKILADCK.....PGTTSGISLK..IYNEQVQFMEQAFKSSLVVDGMGES
PolRSV    GASKAIFEETIKILNDCT.....PGTAGAASLN..KFNEQIKALEAVFG..KIVDDNNAG
SVNaV     EEKNQTLRLIAEILKDMA.....PQMRGVASLE..KYNEQIGIISDIIGVHFEMPGKK..
TCSV      EAQVQKGKEYATILSSCN.....PNAKGSIAME..HYSEHLDKFYAMFGV.........
TNeV      EDLDKLFKQAVKLLSESN.....PGTASSISLK..KYDEQVKYMDKVFSANLSVDDYGEH
TNRV      GDLKKLFESATAILKTSS.....PGTASSISLK..KYSEQIAHLEKAFGSKPVIDDFGSN
TNSV      DQVQELFSSVVKLLGDSN.....PGTAGAISLN..KYNDQLKHMESAFNSKLNIDDYGQN
TYRV      GVSKDIFEETIKILNDCT.....PGTAGAASLN..KFNEQIKALESAFG..KIVDDNGAG
TZSV      EKTQELFTSTVKLLGESN.....PGTAGAISLH..KYNDQLKQMETAFKSKLNVDDFGQN
WBNV      GEMDKMFKSAVELLSKSN.....PGTASSISLK..KYDDQVKYMDRIFNSSLSMDDYGEH
WSMoV     DDLDVMFKQAVKILSESN.....PGTASSISLK..KYEDQVKYMDRIFSANLSVDDYGDH
ZLCV      DAQMEKGKEYANILKACD.....PRLKGSVAME..HYSETLNKFYNMFGV.........
```

```
TSWV      α14  η3
          250

TSWV      ...KK..QAK...LTELA...
ANSV      ...KK..ESK...ISGVA...
BeNMV     .T.SKAKESKNHGFMS.....
CCSV      SK.Q...SSKKKSG...DLTF
CaCV      S..KK..SSKPSTS....IEV
CSNV      ...QK..GSKLIPKDLV....
GBNV      SK.KK..SSKAGPS....LEL
GCFSV     VLNIYLLIVKAFLF.......
GRSV      ...KK..EAK...IAGVA...
GYSV      .....................
HCRV      SS.KPRSASKK...NQGF...
INSV      .D.FG....KNDSI.......
IYSV      SS.KPKTPSKKDRYN......
LNRV      IRGAEGGSSDS..........
MSMV      .S.KKASTSKVFEI.......
MYSV      SS.K....SKASSSRSKSIEV
MVBaV     S..KK..KSQPSTSN..SIEV
PCSV      ...KK..EAK...IAGVA...
PNSV      S..AS..TSKPTKRG..SMS.
PhySMV    SS.K....SKASSSRSKSIEV
PolRSV    SS.KPRPASKK...NQGF...
SVNaV     .D.GKGKKSK..EFSV.....
TCSV      ...RK..EAK...IAGVA...
TNeV      S..KK..SSKLSTS....IEV
TNRV      S..VS..TSKPTRRG..SIG.
TNSV      SK.PG..SSKKPSKL..SL..
TYRV      SS.KPKPSSKK...NDAF...
TZSV      SK.Q...APKKKSSN..DLSF
WBNV      S..KK..SSKASSS....LEV
WSMoV     S..KK..SSKPSTS....LEV
ZLCV      ...KK..EEKHVPKG.VA...
```
