# Supplementary material for: Identification and localization of Tospovirus genus-wide conserved residues in 3D models of the nucleocapsid and the silencing suppressor proteins
Source: Virol J. 2019 Jan 11;16:7. doi: 10.1186/s12985-018-1106-4 (PMC6330412; doi:10.1186/s12985-018-1106-4)
Supplement: Supplementary file 3 — Figure S2. Extended version of the alignment of NSs amino acid sequences of the NSs protein of all known tospoviruses. The secondary structure of TSWV predicted by MULTICOM is shown above the alignment with arrows and squiggles indicating beta sheets and alpha helices, respectively. Amino acid residues conserved among all known tospoviruses are highlighted in red. The figure was prepared using ESPript 3.0 server [40]. (PDF 34 kb) [file 12985_2018_1106_MOESM3_ESM.pdf]

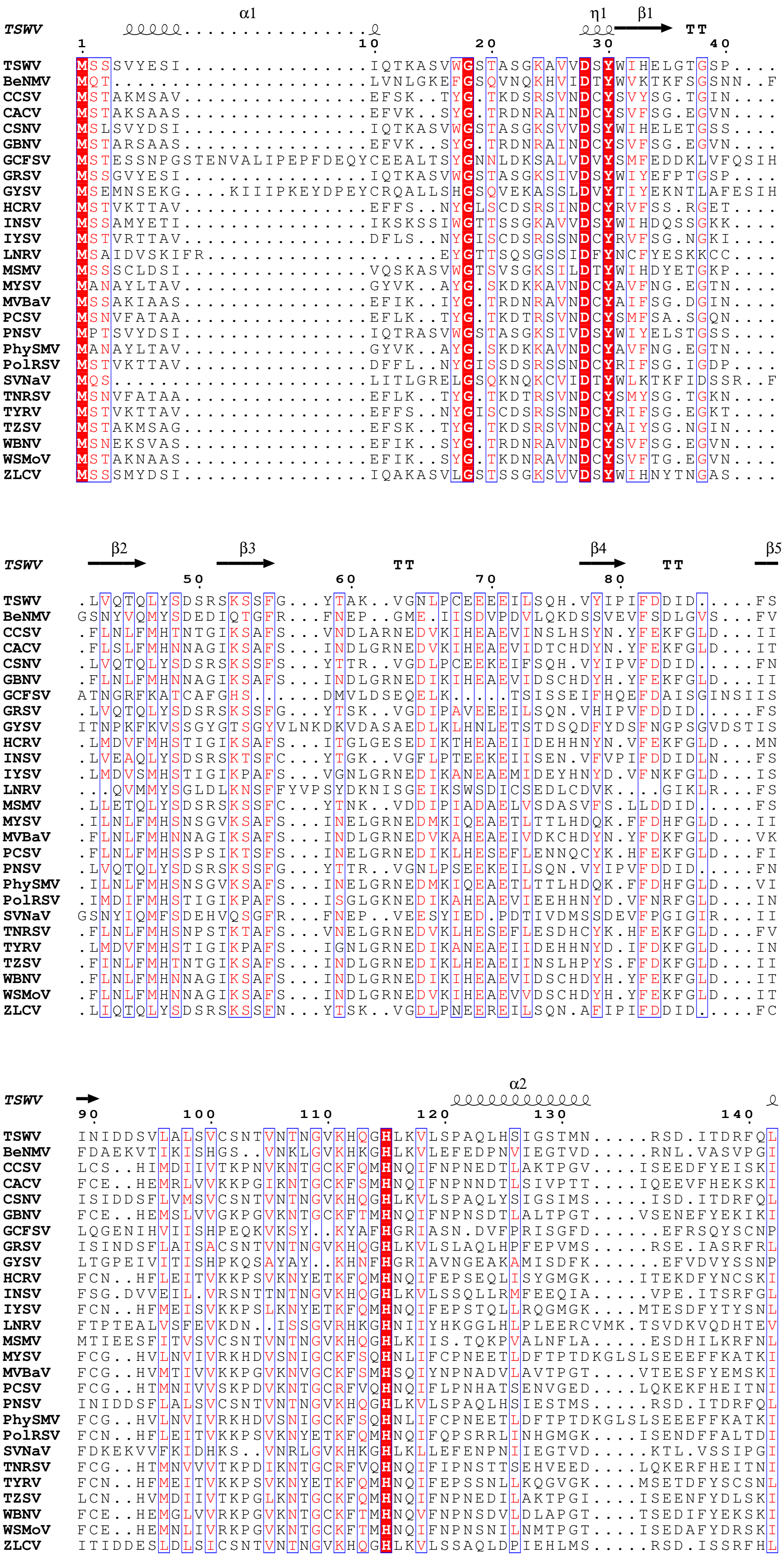

TSWV
α1
η1
β1
TT
1
10
20
30
40
TSWV MSSSVYESI................IQTKASVWGSTASGKAVVDSYWIHELGTGSP....
BeNMV MQT......................LVNLGKEFGSQVNQKHVIDTYWVKTKFSGSNN..F
CCSV MSTAKMSAV................EFSK..TYG.TKDSRSVNDCYSVYSG.TGIN....
CACV MSTAKSAAS................EFVK..SYG.TRDNRAINDCYSVFSG.EGVN....
CSNV MSLSVYDSI................IQTKASVWGSTASGKSVVDSYWIHELETGSS....
GBNV MSTARSAAS................EFVK..SYG.TRDNRAINDCYSVFSG.EGVN....
GCFSV MSTESSNPGSTENVALIPEPFDEQYCEEALTSYGNNLDKSALVDVYSMFEDDKLVFQSIH
GRSV MSSGVYESI................IQTKASVWGSTASGKSIVDSYWIYEFPTGSP....
GYSV MSEMNSEKG....KIIIPKEYDPEYCRQALLSHGSQVEKASSLDVYTIYEKNTLAFESIH
HCRV MSTVKTTAV................EFFS..NYGLSCDSRSINDCYRVFSS.RGET....
INSV MSSAMYETI................IKSKSSIWGTTSSGKAVVDSYWIHDQSSGKK....
IYSV MSTVRTTAV................DFLS..NYGISCDSRSSNDCYRVFSG.NGKI....
LNRV MSAIDVSKIFR....................EYGTTSQSGSSIDFYNCFYESKKCC....
MSMV MSSSCLDSI................VQSKASVWGTSVSGKSILDTYWIHDYETGKP....
MYSV MANAYLTAV................GYVK..AYG.SKDKKAVNDCYAVFNG.EGTN....
MVBaV MSSAKIAAS................EFIK..IYG.TRDNRAVNDCYAIFSG.DGIN....
PCSV MSNVFATAA................EFLK..TYG.TKDTRSVNDCYSMFSA.SGQN....
PNSV MPTSVYDSI................IQTRASVWGSTASGKSIVDSYWIYELSTGSS....
PhySMV MANAYLTAV................GYVK..AYG.SKDKKAVNDCYAVFNG.EGTN....
PolRSV MSTVKTTAV................DFFL..NYGISSDSRSSNDCYRIFSG.IGDP....
SVNaV MQS......................LITLGRELGSQKNQKCVIDTYWLKTKFIDSSR..F
TNRSV MSNVFATAA................EFLK..TYG.TKDTRSVNDCYSMYSG.TGKN....
TYRV MSTVKTTAV................EFFS..NYGISCDSRSSNDCYRIFSG.EGKT....
TZSV MSTAKMSAG................EFSK..TYG.TKDSRSVNDCYAIYSG.NGIN....
WBNV MSNEKSVAS................EFIK..SYG.TRDNRAVNDCYSVFSG.EGVN....
WSMoV MSTAKNAAS................EFIK..SYG.TKDNRAVNDCYSVFTG.EGVN....
ZLCV MSSSMYDSI................IQAKASVLGSTSSGKSVVDSYWIHNYTNGAS....
TSWV
β2
β3
TT
β4
TT
β5
50
60
70
80
TSWV .LVQTQLYSDSRSKSSFG...YTAK..VGNLPCEEEILSQH.VYIPIFDDID.....FS
BeNMV GSNYVQMYSDEDIQTGFR...FNEP..GME.IISDVPDVLQKDSSVEVFSDLGVS...FV
CCSV .FLNLFMHTNTGIKSAFS...VNDLARNEDVKIHEAEVINSLHSYN.YFEKFGLD...II
CACV .FLSLFMHNNAGIKSAFS...INDLGRNEDVKIHEAEVIDTCHDYN.YFEKFGLD...IT
CSNV .LVQTQLYSDSRSKSSFS...YTTR..VGDLPCEEKEIFSQH.VYIPVFDDID.....FN
GBNV .FLNLFMHNNAGIKSAFS...INDLGRNEDIKIHEAEVIDSCHDYH.YFEKFGLD...II
GCFSV ATNGRFKATCAFGHS......DMVLDSEQELK.....TSISSEIFHQEFDAISGINSIIS
GRSV .LVQTQLYSDSRSKSSFG...YTSK..VGDIPAVEEEILSQN.VHIPVFDDID.....FS
GYSV ITNPKFKVSSGYGTSGYVLNKDKVDASAEDLKLHNLETSTDSQDFYDSFNGPSGVDSTIS
HCRV .LMDVFMHSTIGIKSAFS...ITGLGESEDIKTHEAEIIDEHHNYN.VFEKFGLD...MN
INSV .LVEAQLYSDSRSKTSFC...YTGK..VGFLPTEEKEIISEN.VFVPIFDDIDLN...FS
IYSV .LMDVSMHSTIGIKPAFS...VGNLGRNEDIKANEAEMIDEYHNYD.VFNKFGLD...IS
LNRV ...QVMMYSGLDLKNSFFYVPSYDKNISGEIKSWSDICSEDLCDVK....GIKLR...FS
MSMV .LLETQLYSDSRSKSSFC...YTNK..VDDIPIADAELVSDASVFS.LLDDID.....FS
MYSV .ILNLFMHSNSGVKSAFS...INELGRNEDMKIQEAETLTTLHDQK.FFDHFGLD...II
MVBaV .FLNLFMHNNAGIKSAFS...INDLGRNEDVKAHEAEIVDKCHDYN.YFDKFGLD...VK
PCSV .FLNLFMHSSPSIKTSFS...INELGRNEDIKLHESEFLENNQCYK.HFEKFGLD...FI
PNSV .LVQTQLYSDSRSKSSFG...YTTR..VGNLPSEEKEILSQN.VYIPVFDDID.....FN
PhySMV .ILNLFMHSNSGVKSAFS...INELGRNEDMKIQEAETLTTLHDQK.FFDHFGLD...VI
PolRSV .IMDIFMHSTIGIKPAFS...ISGLGKNEDIKAHEAEVIEEHHNYD.VFNRFGLD...IN
SVNaV GSNYIQMFSDEHVQSGFR...FNEP..VEESYIED.PDTIVDMSSDEVFPGIGIR...II
TNRSV .FLNLFMHSNPSTKTAFS...VNELGRNEDVKLHESEFLESDHCYK.HFEKFGLD...FV
TYRV .LMDVFMHSTIGIKPAFS...IGNLGRNEDIKANEAEIIDEHHNYD.IFDKFGLD...IN
TZSV .FINLFMHTNTGIKSAFS...INDLGRNEDIKLHEAEIINSLHPYS.FFDKFGLD...II
WBNV .FLNLFMHNNAGIKSAFS...INDLGRNEDIKIHEAEVIDSCHDYH.YFEKFGLD...IT
WSMoV .FLNLFMHNNAGIKSAFS...INDLGRNEDVKIHEAEVVDSCHDYH.YFEKFGLD...IT
ZLCV .LIQTQLYSDSRSKSSFN...YTSK..VGDLPNEEREILSQN.AFIPIFDDID.....FC
TSWV
α2
90
100
110
120
130
140
TSWV INIDDSVLALSVCSNTVNTNGVKHQGHLKVLSPAQLHSIGSTMN.....RSD.ITDRFQL
BeNMV FDAEKVTIKISHGS..VNKLGVKHKGHLKVLEFEDPNVIEGTVD.....RNL.VASVPGI
CCSV LCS..HIMDIIVTKPNVKNTGCKFQMHNQIFNPNEDTLAKTPGV....ISEEDFYEISKI
CACV FCE..HEMRLVVKKPGIKNTGCKFSMHNQIFNPNNDTLSIVPTT....IQEEVFHEKSKI
CSNV ISIDDSFLVMSVCSNTVNTNGVKHQGHLKVLSPAQLYSIGSIMS.....ISD.ITDRFQL
GBNV FCE..HEMSLVVGKPGVKNTGCKFTMHNQIFNPNSDTLALTPGT....VSENEFYEKIKI
GCFSV LQGENIHVIISHPEQKVKSY..KYAFHGRIASN.DVFPRISGFD......EFRSQYSCNP
GRSV ISINDSFLAISACSNTVNTNGVKHQGHLKVLSLAQLHPFEPVMS.....RSE.IASRFRL
GYSV LTGPEIVITISHPKQSAYAY..KHNFHGRIAVNGEAKAMISDFK......EFVDVYSSNP
HCRV FCN..HFLEITVKKPSVKNYETKFQMHNQIFEPSEQLISYGMGK....ITEKDFYNCSKI
INSV FSG.DVVEIL.VRSNTTNTNGVKHQGHLKVLSSQLLRMFEEQIA.....VPE.ITSRFGL
IYSV FCN..HFMEISVKKPSLKNYETKFQMHNQIFEPSTQLRQGMGK....MTESDFYTYSNL
LNRV FTPTEALVSFEVKDN..ISSGVRHKGHNIIYHKGGLHLPLEERCVMK.TSVDKVQDHTEV
MSMV MTIEESFITVSVCSNTVNTNGVKHQGHLKIIS.TQKPVALNFLA.....ESDHILKRFNL
MYSV FCG..HVLNVIVRKHDVSNIGCKFSQHNLIFCPNEETLDFTPTDKGLSLSEEEFFKATKI
MVBaV FCG..HVMTIVVKKPGVKNVGCKFSMHSQIYNPNADVLAVTPGT....VTEESFYEMSKI
PCSV FCG..HTMNIVVSKPDVKNTGCRFVQHNQIFLPNHATSENVGED....LQKEKFHEITNI
PNSV INIDDSFLALSVCSNTVNTNGVKHQGHLKVLSPAQLHSIESTMS.....RSD.ITDRFQL
PhySMV FCG..HVLNVIVRKHDVSNIGCKFSQHNLIFCPNEETLDFTPTDKGLSLSEEEFFKATKI
PolRSV FCN..HFLEITVKKPSVKNYETKFQMHNQIFQPSRRLINHGMGK....ISENDFFALTDI
SVNaV FDKEKVVFKIDHKS..VNRLGVKHKGHLKLLEFENPNIIEGTVD.....KTL.VSSIPGI
TNRSV FCG..HTMNVVVTKPDIKNTGCRFVQHNQIFIPNSTTSEHVEED....LQKERFHEITNI
TYRV FCN..HFMEITVKKPSVKNYETKFQMHNQIFEPSSNLLKQGVGK....MSETDFYSCSNL
TZSV LCN..HVMDIIVTKPGLKNTGCKFQMHNQIFNPNEDILAKTPGI....ISEENFYDLSKI
WBNV FCE..HEMGLVVRKPGVKNTGCKFTMHNQVFNPNSDVLDLAPGT....TSEDIFYEKSKI
WSMoV FCE..HEMNLIVRKPGVKNTGCKFTMHNQIFNPNSNILNMTPGT....ISEDAFYDRSKI
ZLCV ITIDDESLDLSICSNTVNTNGVKHQGHLKVLSSAQLDPIEHLMS.....RSD.ISSRFHL

```
            α3            β6        β7              β8
TSWV                                         TT                          TT
                  150          160          170         180                 190
TSWV     QEKDIIPNDRYIEAVNKGSLSCVKEHAYKIEMCYNLALGKVN...........VLSPNR.
BeNMV    NNDDIEIKNETINALPSMNMIHLKEDSYKITKSPNCFFGKRN...........MLR..RG
CCSV     KLKGLTPFGWYVDECKKHDFYISNSGDISLDYG.FPVMGKT.............TSYWRE
CACV     RSNDLLPSDWCLDEFWKNNFYIATNGEFSLDYG.FSVMGKT.............TSYWRE
CSNV     QEKDIIPNDRYIEAANKGSLSCVKESTYEVKMCHNQALGKVN...........VLSPNR.
GBNV     RSNGLLPSGWYQDECCKNNFYIATNGDLTLDYG.FSVMGKT.............TSYWRE
GCFSV    YKFVYNPGMLGLESETN.TVIFPVEKLGTLPAH.ASAFGNNFDRCHFSGSSVP.......
GRSV     QEEDIIPDDKYISAANKGSLSCVKEHTYKVEMSYNQALGKVN...........VLSPNR.
GYSV     YNFLYNPKILNIE.ETSSTILFPVEQIGTLPST.ISVFGRYFDKNHFSVVDAN.......
HCRV     SPEDIYPSDWFINEAKKKNFYMADISGFSMDWG.FSVMGRT.............TSYWKE
INSV     KESDIFPPNNFIEAANKGSLSCVKEVLFDVKYSNNQSMGKVS...........VLSPTR.
IYSV     SKDEIYPNEWFVSEAKRKNFFIADVSGFSLDYG.FSVMGKT.............TSYWKE
LNRV     SPEAFLPSFHTINEALKSNVITLDTECFALDGRKVGYLGTYNG........RKYVS....
MSMV     KETDIVPEDRFIAAANRGSLSCVKEIMYETKTSNNQAYGKVN...........VLSPNR.
MYSV     TSEDFHPQGWCIEECKKNRFTVAETSCLELNYG.SPVMGKT.............VAYWRE
MVBaV    KPNGLGPAEWYLEECKRNNFYVASNGDVTIDYG.FSVMGKT.............TSYWRE
PCSV     KNYCLAPNAWVIELCLRSNFFISASGDYKIEYG.YPVMGKT.............VSYWRE
PNSV     QEKDIIPDDRYIEAANKGSLSCVKEHVYKVEMCNNQALGKVN...........VLSPNR.
PhySMV   TSEDFHPQGWCFEECKKNRFTVAETSCLELNYG.SPIMGKT.............VAYWRE
PolRSV   AKEDIYPRDWFINEAERKTFFMADIAGFSLDYG.FSVMGKT.............TSYWRE
SVNaV    CAEDITVDDELTGLLPSMNITHMKEDSYKITKSNNCFFGKRN...........MMR..RG
TNRSV    KTNTLTPSAWATELCIRNNFFISMGGNYRLEYG.YPVMGKT.............VSYWRE
TYRV     SKDDMYPSEWFINEARRKNFFMADVSGFNLDYG.FSVMGKT.............TSYWKE
TZSV     KPKGMTPFDWYINECKKYDFYISDNGDISLDYG.FPVMGKT.............TSYWRE
WBNV     RPIGMTPSGWCLDECRRNNFYIAANGELALDYG.FSVMGKT.............TSYWRE
WSMoV    ASSGLLPSRWCLDECYKSNFYIATNGDLTLDYG.FSVMGRT.............TSYWRE
ZLCV     KEEDIIPKDRYIEAANRGSLSCVKEQTYEVKVCHNQALGKVN...........VLSPNR.

                               β9          β10
TSWV                                    TT
                       200                210         220          230
TSWV     ........NVHEWLYSFKPN....FNQVESNNRTVNSLAVKSLLMSAENNIMPN..SQAF
BeNMV    WIGTGYVTNIHPIGYVL.P.........KATNRCLGTLSLKSMESTVEKGVL....SSDI
CCSV     N.......LPREKIISVKQKCV..PNVSALTNRILPLPTVKAIQIASE..LASE..KTVV
CACV     N.......ISREKILSFKHKSL..PDNTVPTNRLLSTSIVKGIQLGSE..LAPE..TTVI
CSNV     ........NVHEWLYSFKPN....FNQVESNNRTVNSLAVKSLLLSAENNIMPN..SQAF
GBNV     S.......ISREKILSVKQKCL..PDNTVPTNRLLSTSTVKGIQLGSE..LAPD..TTVI
GCFSV    ............GFMTVKAVSESSKRTQEKAHRQLHNKSLKALEIVSNSDFRSTYITKTT
GRSV     ........NVHEWLYSFKPN....FNQIESNNRTVNSLAVKSLLMATENNIMPN..SQAF
GYSV     ............KYMSVKAVSDGAKYSNQGSHRQLHNKNLKAIEIASVSGIMPNTISKTT
HCRV     N.......MEKTSLVSVKQKSA..AFPSVPTNRVLSVSTIKAVEIVSK..IACD..KSTI
INSV     ........SVHEWLYTLKPV....FNQSQTNNRTVNTLAVKSLAMSATSDLMSD..THSF
IYSV     N.......MDKTSLISVKQKSM..NNPSVPTNRLLSASTIKAIEIASN..IACN..KSTV
LNRV     ........SQGFYMASVKPISAVSKG..KNSNRLLSAHILKSVEDFCEMGCTHT....QV
MSMV     ........NSNEWIYTVKPM....FNQTETNNRTVNSLAIKSLLLSAVNDISPC..SQVF
MYSV     N.......ISGEKLVTVKPKCI..NVPTALPNRLLPSSTVKAIQIGSE..LAKQ..ATVV
MVBaV    N.......ISREKILSVKQKSL..SDSTVPSNRLLSTSTIRAIQLGSD..LAPE..NSTI
PCSV     N.......LPKGKMLSIKQKVA..QGTSRLTNRVLSPSAARAIQIAAE..IVQN..ENTI
PNSV     ........NVHEWLYSFKPI....FNQIESNNRTVNSLAVKSLLMSAENNIMPN..SQAF
PhySMV   N.......ISGEKLVTVKPKCI..NVPTALPNRLLPLSTVKAIQIGSE..LAKQ..ATVV
PolRSV   N.......LEKTSLISIKQKSA..GNPVVPTNRLLSAGTIKAVEIASA..IASD..KTTI
SVNaV    WIGTGNVTSIHPVGYSM.P.........ASTNRCIGTLALKSLESTAKKGIR....STDI
TNRSV    N.......LQKEKMLSVKQKVI..QGTSRLTNRVLSPSAVKAIQIASE..LVQS..ETTI
TYRV     N.......MDKTSLISVKQKSM..NNPLVPTNRLLSASTIKAIEIASN..IACN..KSTI
TZSV     N.......IPREKIISVKQKCL..PNTSALTNRILSLSTVRAIQIASE..LASE..KTVV
WBNV     N.......MSREKILSVKQKSL..PDNTVPTNRLLSTSTVKGIQLGSE..LASD..NTVI
WSMoV    S.......MSREKILSVKQKCF..SDNTVPTNRLLSTSTVKGIQIGSE..LASD..TTVI
ZLCV     ........NVHECLYSFKPN....FNQVESNNRTVNSLAIKSLLMSVKNEIMPN..SQAF

           η2      β11             η3                            β12
TSWV                                                          TT
          240        250          260        270        280        290
TSWV     VKASTDSHFKLSLWLR..VPK.VLKQISIQKLFKVAGDETN.KTFYLS..IACIPNHNSV
BeNMV    AHAELSLGGKCNFHLFS.SCKGDLGDVSFMRNVKFFEDKHERVFTIYVKTSSDIDN.N..
CCSV     LASRQRLDIDVKSQYRV.SFPGIQDEGAFTRTFCIPMDNTARIICFYAKTSVDSSN....
CACV     LSCKQNLDIDLKSQYRI.SFHGIQEEGAFTRTFCIPFENKSRIICFYAKTVADNSN....
CSNV     VKASTDSHFKLTLWLR..IPK.VLKQISSQRLFKIAGDETN.KEFYFS..IACIPNHNSV
GBNV     LSLKQNLNVDLKSQYRV.SFHGIQEEGAFARTFCVPFENKSRMICLYAKTVVDNSN....
GCFSV    LKARKNGMMTIQVQVK..ISTASLK.....REHLIAIPDDP.....LKRLVYCSSECLGQ
GRSV     VEASTDSHFRLSLWLR..IPK.VLKQIAIQKLFRFAGDETG.KSFYLS..IACIPNHNSV
GYSV     MQTKKNCTVYIQVQVLD.PSCKPEK.....KECLIQAKDDP.....YQRLVYCKSECLGQ
HCRV     LSVRQDLRSDLRTQFRI.SLPGEYSDTSVARTYLLHQGSKGQYICIYARTTMDSPN....
INSV     VRLNNNKPFKISLWMR..IPK.IMKSNTYSRFFTLS.DESSPKEYYIS..IQCLPNHNNV
IYSV     LAVKQDLSFDLKTQFRI.SFPGEYNETAIARTFLINQGMKGQYICIYAKTVMDKSN....
LNRV     MSAKVSLNNDFTFVNYINKIEPTFEEYEIVKHFKYHKDKSARFFTITFSMFRDFDGVHN.
MSMV     LKAFTDKSFKLSFWLR..IPK.VLKHTDFYKCFKIPNGSSN.....LSLTINCIPNYNNI
MYSV     LSARQSLSQDLKSQYRV.TFPGVLEDLAYSRSYLVPFGAKNRIIRFDSRTISDREN....
MVBaV    LSCRQNLGLDLKSQYRV.SFPGIQEEGAFAKTFCVPCGSKLRMIYFHAKTVADNSN....
PCSV     LSVRQLLTEDIKSQFRI.CFTGALEEGSFTRTYKIRAGQEDRVICIYAKTIIDSSY....
PNSV     VKASTDSHFKLSLWLR..IPK.VLKQIAIQKFFTVAGDETG.KSFYLS..IACIPNHNSV
PhySMV   LSARQSLSQDLKSQYRV.TFPGVLEDLAYSRSYLVPFGAENRIIRFDSRTISDREN....
PolRSV   LAVKQDLDADLRTQFRI.SLPGEYNEAGLARTFLINQGSKGQYICIYARTSLDRPN....
SVNaV    SHAEIDINGLCNFHLFT.SCKGDLGDKAWMKTLKFFHDDHERVFSIYIRTTSDIDN.N..
TNRSV    LSARQLLTENIKSQYRI.CFNSVLEEGSFTRTYKVRAGQDVRVICIYAKTVIDSSY....
TYRV     LAVKQDLTSDLKTQFRI.SLPGEYNETAIARTFLINQGKKGQYICIYAKTVMDRSN....
TZSV     LASRQRLDLDIKSQYRI.SFPGVQDEGAFTRTFCIPMDKTARIVCFYAKTSVDVSN....
WBNV     LSLKQNLNIDLKSQYRI.SFHGIQEEGAFARTFCVPFENKSRMICMYAKTVVDNSN....
WSMoV    LSCKQNLNIDLKSQYRI.SFHGIQEEGVFARTFCVPFENKSRMISFYAKTVADNSN....
ZLCV     ASISTDADFKVNFWLR..IPR.VLKQVNFQKLFKIAEDTSN.KDFYLS..IACIPSHSSV
```

```
                      β13                      β14
TSWV
                 300                  310                 320          330
TSWV     ETALNISVICK..........HQLPIRKCKAP..FEL.......SMMFSDLKEPYNIVHD
BeNMV    ..KSSILVIFKTVCYSE.SK.EGT..VSVSSDDKLQG.......SINLSELPESG..FFD
CCSV     ...ERTTLTIKIVN....KTIENNCPGPIPKDHIYCD.......KSIGARIGLVDVVRGD
CACV     ...ERTTLIIKVVT....KTIDSHLMIPIRN.HINCS.......RLVGARIGLVDFCDAD
CSNV     ETALNITVICK..........HQLPIPKIKAP..FEL.......SMMFSDLKEPYNIVHD
GBNV     ...ERTTLIIKVVT....KTIESHLVIPIRN.HINCV.......KKIGARIGLVDSIESD
GCFSV    TSPGLSTFIFEVMCLDCLVDRVTPDVTYFAGALSTCFTDEPPHILLKARSGILSSLADSD
GRSV     ETALNVTVICK..........HQLPIPKSKAP..FEL.......SMIFSDLKEPYNTVHD
GYSV     TKPGISTFLFKIICLDCTQSEVSHPTTYFAGALSTCLDLEEMPSLVRPASANLNAMVDTD
HCRV     ...ERTTLIIKIFTQ...SKPGGFTTTLLPNDHSNCR.......KMVGASFGIVEQKMTD
INSV     ETALNITLISK..........LLLESTPSSSD..HKI.......EMNFSDLKEPYNVIHD
IYSV     ...ERTTLILKIVTQ...NRPGSYNSTLLPKNHSDCR.......KVVGASFGIVEQKSTD
LNRV     ...TYKTSISYKNPSR......MLDTTAQVSEKSASI..........ASLVIGDVNVED
MSMV     ETALNFSLVS.............LKPKEPLSAPKQGKF......VINFSSLKEPCCNVFD
MYSV     ...EQTTLILKTST....KTIESN.FINRAQEHKNCI.......KSIGERLGLVDFIVSE
MVBaV    ...ERTTLIIKVVT....KTVDSGLSIPARN.HITCD.......MTIGARIGLVGFVNDD
PCSV     ...ESTTLIVKVVN....KSVKSDHHEVLQN.HSDC........KSISSSLGITDSFNGD
PNSV     ETALNVTVICK..........HQLPITKLKAP..FEL.......SMMFSDLKEPYNIVHD
PhySMV   ...EQTTLILKTST....KTIESN.FINRAQEHKNCI.......KSIGERLGLVDFVVSE
PolRSV   ...ERTTLIIKVVTQ...KRPGDYDMMMLPRDHANCR.......KLVGASFGTVERKLTD
SVNaV    ..ISNILLIFK.VLR...FKRFATPKVVVSQDDDSAA.......EINLSEGSASG..CFD
TNRSV    ...ESTTLIIKIVN....KSISNDYSDVLPV.HSDC........KYVSSRLGITDVFNGD
TYRV     ...ERTTLIIKVVTQ...SRPGEYISTLLPKDHSDCR.......RVVGASFGIVEQKPTD
TZSV     ...ERTTLTIKIVN....KTVESNYMGPVPKDHMYCD.......KSIGARIGLVDIVRGD
WBNV     ...ERTTLIIKVVT....KTVDSHLVIPIRN.HANCT.......RKIGARIGLVDFCDTD
WSMoV    ...ERTTLIIKVVT....KTVDSHLIIPIRN.HINCE.......KKIGARVGLVDFCDSD
ZLCV     ETALNVTIICK..........HLKPTPEILSF..FEL.......KMAFSDLKEPYSAVHD
```

```
                                                     β15       β16
TSWV                                        TT
                340       350       360       370       380
TSWV     P....SYPQRIVHALLETHTSFAQVLCNNLQEDVIIYTLNNH.ELTPGKLDLGE.RTLNY
BeNMV    HK...AKSMLIVQSLIKIHNEFASKISEKLNKSVIVYTLRSV.DSFKKEITEIDSRAMNY
CCSV     P....NYNLMIAREMVSVHTNFALKLSESLKKPVIVFKMYDK.ELNFETYDLSG.RSLSY
CACV     P....NYNQMIVKELLGVHTQFAMNLSGALKKPIIVFKMYDK.ELINGHVEVSG.RVLNY
CSNV     P....SYPQRIVHGLLETHTSFAQTLSNNLQEDVIIYSLNNH.ELTPAKLDLGD.RTLNY
GBNV     P....NYNQMIVKELLGVHTQFAINLSLVIKKPIIVFKMYDI.ELNNNHIDVYG.RILNY
GCFSV    RD...KINEIICKNLVSVHLGLATDLAKKLNKPINVFTIKDTSSMTTDMVEVDG.KKYRV
GRSV     P....SYPQRIVHALLETHTSFAQVLCNKLQGDVIIYTINSP.ELTPAKLDLGE.RTLNY
GYSV     RE...KINEIICKNLVAVHLELAQDISKKLNKPINVFTIKDTSNMTGDTVDVNG.KKFRI
HCRV     P....NYNKIIAHELLSVHTNFALKISKVLQKPVIVYKVYEK.ELPPKRVEIDG.RTFNY
INSV     M....SYPQRIVHSLLEIHTELAQTICDSVQQDIIVFTINEP.DLKPKKFELGK.KTLNY
IYSV     P....NYNKIIANELLSVHTNFALKISKILKKPVIVYKTYDK.ELLPKKVEIDG.RTFNY
LNRV     PIKFQDIDSKIVGALMEIHLGLAGKLRESIKKDIVVYTKQEE....PLDVILDPQTNLRY
MSMV     L....TYPQRIVHSLLEVHTSLAKKLSDFLREEVIIYTLNLP.ETEVKKLDLAG.RTLNY
MYSV     P....NYSQIIASDFLRLQTVLSLRVSKHFKKPVIVYKLYDK.ETELKKAKIDG.KFVSY
MVBaV    L....NYNQMIAKELIAVHTSFALKLSEILKKPVIVFKMYDK.ELEYDSANLME.RALSY
PCSV     P....NYNQIIARSLIKTHTLFALEISKYLEKKVIIFMLYEK.QLTKKTMPYPV.RDLSY
PNSV     P....SYPQRIVQALLETHTSFAQVLCNNPQEDVIIYTLNKH.ELTPAKLDLGD.KTLNY
PhySMV   P....NYSQIIASDFLRLQTVLSFRVSKHFKKPVIVYKLYDK.ETELKKVKIDG.KFVSY
PolRSV   P....NYNKIIAHEMLAVHTSFALKISKILSKPVIVYKVYEK.ELSPKAVEVDG.RTFNY
SVNaV    LR...TKSVVIVQSLMRIHNEFALSLSKFLKKKVIVYTLRDS.LSFTKKVAEIDSRFLNY
TNRSV    P....NYNQIIARSLVNMHTLFALELSKHLRKKVIVFTLYEK.QLTKKTMPSPT.RDLSY
TYRV     P....NYNKIIAHELLSVHTNFALKISKVLKKPVIVYKIYDK.ELLPKKVEIDG.RTFNY
TZSV     P....NYNQMIAREMISVHTNFALRLSEALKKPVIVFKMYEK.ELNFETYDLSG.RSLSY
WBNV     P....NYNQMIVKELLSVHTQFAIKSSEVVGKPIIVFKMYDK.ELSNNHVDVFG.RILNY
WSMoV    P....NYNQLIVKNLLSVHTQFAINLSEVIQKPVIVFKMYDK.ELHNDYTEVSG.RILNY
ZLCV     P....SYSHRIAHALLETHTSFAQSLCDKLQEDVIIYSLNNH.ELFPMKLDVGG.RTLNY
```

```
                   β17       α4         β18         β19     β20     β21
TSWV                                           TT
           390       400        410        420        430        440
TSWV     SEDICKRKYFLSKT.LECLP...SNTQTMS.YLDSIQIPSWKIDFARGEIKISPQSVSVT
BeNMV    LISPEGEVFFLSRTFQESLP...TNMSSLT.YFFDLQTFGWSANYLSG............
CCSV     QKDSSGNIYFLSKT.LEILP...KSLSTLT.YLKSISPACWKESISMQHFYVGEQEGESS
CACV     QVDADGNIYFLSKT.LEVLP...RSLSTLS.YLNSVAPTLWKESLEHQHFIV........
CSNV     SEDAYKRKYFLSKT.LDCLP...VNTQTMS.YLDSIQIPSWKVDFARGEIKISPQSISVA
GBNV     QTDAEGNAYFLSKT.LEVLP...KSLSTLS.YLGSIAPIQWEESLEHQHFIV........
GCFSV    LKDSNGDYYFTSATFEKTLIGAYKSCQTFVDYCDN......KKLSINGDNVFIFN.....
GRSV     SEDASKKRYFLSKT.LECLP...VNVQTMS.YLDSIQIPSWKIDFARGEIRISPQSTPIA
GYSV     LKDSSGDCYFTSATFEKTFIGAYKSCQTFIDYCDS......KKLSINGDNVFI.......
HCRV     QEDIDGNIYFLSTT.LAILP...LSLSVLS.YLDSASPPCWKESKGLGHFTVEEIQYI..
INSV     SEDGYGRKYFLSQT.LKSLP...RNSQTMS.YLDSIQMPDWKFDYAAGETKISPRSEDVL
IYSV     QEDIDGNIYFLSTT.LAILP...VSVSVLS.YLDSASPSCWKESKGLGHFTVEELQ....
LNRV     YVKGE.NAYFLSKTFQNALP....VDHALN.YFIS...KAAKAIVIKGGDIFLSASKD..
MSMV     NESPSGKKYFLSQT.LNCLP...KNSQSLA.YLNSFQFCSLHVDYIRGEICVLPTLSSAS
MYSV     NEDSEGNVYVLNKT.LDLFP...KSNSSFV.YLSRTMSPFWKEFPFEQHLVVDYNEFLER
MVBaV    QKDAEGNIYFLSKT.LDILP...KSPSTLI.YLNSIVPNYWKESAEGQHYTV........
PCSV     LEDSDGNVYFTSET.LKLLP...KSLSTIT.YLKGIAPSCWKESIENQHFYVEYKQP...
PNSV     SEDAYKKKYFLSKT.LECLP...ANTQTMS.YLDSIQIPSWKIDFARGEIKISPLAVSVT
PhySMV   NEDSEGNVYVLNKT.LDLFP...KSNSSFM.YLSKTMSPFWKEFPFEQHLVVDYNEFSER
PolRSV   NEDVDGNVYFLSKT.LEMLP...ISLSVLV.YLDSAAPPCWKEHRGLGHFTVEEVQ....
SVNaV    LEDSDGNIYLLSRTFQESLP...LNRDSLS.YFFDLQTFGWAANHAAG............
TNRSV    LEDSDGNVYFTSET.LKTLP...KSISAII.YLKGVAPCCWRESIEDQHFYVEYRPP...
TYRV     QEDIDGNVYFLSTT.LAILP...LSVSVLF.YLESASPSCWKESRGLGHFTVEEVQ....
TZSV     QKDSSGNIYFLSRT.LEILP...KSLSTLT.YLKNISPACWKESISMQHFYIGEQE.EGP
WBNV     QTDTEGNIYFLSKT.LEVLP...KSLSTLS.YLGSIAPIQWIECLEHQHFVV........
WSMoV    QVDSEGNIYFLSKT.LEVLP...KSLSILS.YLGSIAPIQWKECLEHQHFVV........
ZLCV     SEDAYKRKYFISET.LECLP...ANMQTIS.YLESIQIPSWKIDFARGEIKISPRSPSFL
```

```
                   β22            β23
TSWV      ►TTT--->          --->
                      450            460
                        .              .
TSWV      KSLLKLDLSGIKKEKSKISEAY..ASGSK
BeNMV     ......DLVVRK.................
CCSV      SGSDLAEFEEKSEDA..............
CACV      ......ELK....................
CSNV      KSLLALDLSGIGKKKSKAPAGS..QPEPK
GBNV      ......KPE....................
GCFSV     .............................
GRSV      RSLLKLDLSKIKEKKSLIWETSSYDLE..
GYSV      .............................
HCRV      .............................
INSV      KAISKLDLN....................
IYSV      .............................
LNRV      .............................
MSMV      RANLRLDIASTMS.KSPF...........
MYSV      YLNSSSDSEEEPSTSSTSSTS........
MVBaV     ......EFK....................
PCSV      .....AISEGTTSGTHS............
PNSV      RSLLTLDLSGAEKKKSQLEEAS..KLESK
PhySMV    YLNSSSDSEEEPSTSSTSSTS........
PolRSV    .............................
SVNaV     ......DLATYFE................
TNRSV     .....ADSTETASG.SS............
TYRV      .............................
TZSV      SGSNLAESEEKVQTA..............
WBNV      ......IAN....................
WSMoV     ......KAE....................
ZLCV      KWLVKLDIDEIGRRRPRFSEAH..KSGSK
```
