# Supplementary figures and images for: Identification and localization of Tospovirus genus-wide conserved residues in 3D models of the nucleocapsid and the silencing suppressor proteins
Source: Virol J. 2019 Jan 11;16:7. doi: 10.1186/s12985-018-1106-4 (PMC6330412; doi:10.1186/s12985-018-1106-4)

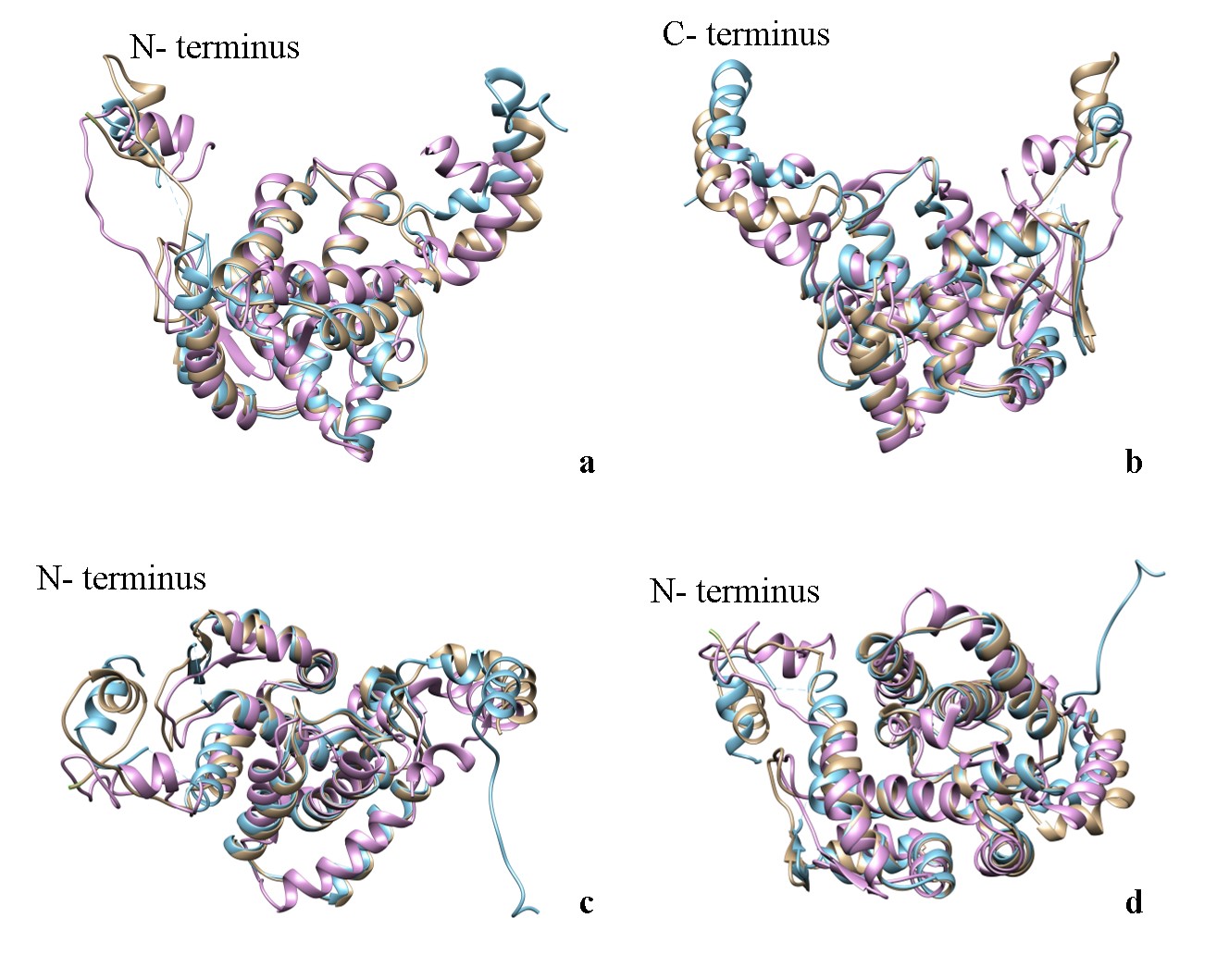

Supplement: Supplementary file 4 — Figure S3. Superimposed model of TSWV N protein crystal structure from Komoda et al. [37] -5iP1 chain A- (brown color), Guo et al. [38] -5y6j chain A- (blue color) and I-TASSER prediction model (This study) (purple color). A) front view, B) rotate 180° horizontal, C) rotated 90° vertical (upper view), D) rotated 270° vertical (bottom view). (JPG 240 kb) [file 12985_2018_1106_MOESM4_ESM.jpg]
